# Assessing the Role of Tandem Repeats in Shaping the Genomic Architecture of Great Apes

**DOI:** 10.1371/journal.pone.0027239

**Published:** 2011-11-04

**Authors:** Marta Farré, Montserrat Bosch, Francesc López-Giráldez, Montserrat Ponsà, Aurora Ruiz-Herrera

**Affiliations:** 1 Departament de Biologia Cel·lular, Fisiologia i Immunologia, Universitat Autònoma de Barcelona, Cerdanyola del Vallès, Spain; 2 Genètica de la Conservació Animal, IRTA, Cabrils, Spain; 3 Department of Ecology and Evolutionary Biology, Yale University, New Haven, Connecticut, United States of America; 4 Institut de Biotecnologia i Biomedicina (IBB), Universitat Autònoma de Barcelona, Cerdanyola del Vallès, Spain; University of Wyoming, United States of America

## Abstract

**Background:**

Ancestral reconstructions of mammalian genomes have revealed that evolutionary breakpoint regions are clustered in regions that are more prone to break and reorganize. What is still unclear to evolutionary biologists is whether these regions are physically unstable due solely to sequence composition and/or genome organization, or do they represent genomic areas where the selection against breakpoints is minimal.

**Methodology and Principal Findings:**

Here we present a comprehensive study of the distribution of tandem repeats in great apes. We analyzed the distribution of tandem repeats in relation to the localization of evolutionary breakpoint regions in the human, chimpanzee, orangutan and macaque genomes. We observed an accumulation of tandem repeats in the genomic regions implicated in chromosomal reorganizations. In the case of the human genome our analyses revealed that evolutionary breakpoint regions contained more base pairs implicated in tandem repeats compared to synteny blocks, being the AAAT motif the most frequently involved in evolutionary regions. We found that those AAAT repeats located in evolutionary regions were preferentially associated with *Alu* elements.

**Significance:**

Our observations provide evidence for the role of tandem repeats in shaping mammalian genome architecture. We hypothesize that an accumulation of specific tandem repeats in evolutionary regions can promote genome instability by altering the state of the chromatin conformation or by promoting the insertion of transposable elements.

## Introduction

Since the earliest cytogenetic studies, evolutionary biologists have sought to understand how mammalian genomes are organized. The characterization of orthologous chromosomal segments among several mammalian species was initially performed by means of G-banding comparisons [1], [2]. Advances in molecular cytogenetic techniques, such as cross-species in situ hybridization, increased the level of resolution for defining orthologous regions as well as the number of species studied [3]. As a result, the integration of cross-species chromosome painting studies performed in more than 100 mammalian species [4], [5] has revealed that evolutionary breakpoints (i.e., the disruption of two orthologous chromosomal segments) are not homogeneously distributed but rather concentrated in certain regions across the human genome.

The multiple ongoing genome sequencing projects are producing an extraordinary amount of data to further refine genome comparisons at a deeper level of resolution: the DNA sequence level. The public availability of these data makes it possible to establish reliable comparisons among genomes, thus providing new insights into the driving forces that generate gene variation, adaptation and evolution. Different approaches have been developed in order to define homologous synteny blocks (HSBs; i.e. regions where the gene order has been conserved among species) and evolutionary breakpoint regions (EBRs; i.e. regions where the synteny has been disrupted by chromosomal reorganizations) among mammalian genomes. Early studies were based on pair-wise comparisons between human and mouse or human and rat genomes [6], [7], using the human genome as a reference whereas recent approaches have gone even further by establishing pair-wise comparisons among several vertebrate species [8]–[11].

Confirming previous cytogenetic studies, in silico analysis lead to the fragile-breakage model, founded initially on mathematical algorithms [6], [12]. According to this model, EBRs are located in specific regions and have been used repeatedly during evolution (i.e., “reused”). In a phylogenetic context, the term “breakpoint reuse” accounts for the recurrence of the same breakpoint in two different species, but not in the common ancestor, based on comparison with an outgroup lineage [8], [11], [13]. The assumption that some chromosome regions have been reused during mammalian chromosomal evolution leads evolutionary biologists to investigate whether there is any particular DNA configuration or composition driving genome instability. Are these evolutionary regions physically unstable due to sequence composition and/or genome organization, or do they merely represent genomic areas where the selection against breakpoints is minimal?

An interesting aspect that has emerged from comparative genomic studies is the finding that breakpoint regions are rich in repetitive elements, for example tandem repeats [14], segmental duplications [15]–[17], and transposable elements [18]–[20]. Repetitive elements represent nearly 50% of the human genome [21]. Among them are tandem repeats, which consist of perfect (or slightly imperfect) copies of a motif in a head to tail fashion, and comprise about 3% of the human genome [21]. They can be classified into two groups, microsatellites and minisatellites. Microsatellites are short tandem repeats with 1-6 bp as a repeat unit, whereas minisatellites contain repeat units ≥7 bp [22]. Tandem repeats have been regarded as an important source of DNA variation and mutation [23]. Tandem repeats can form non-B DNA structures (i.e., DNA structures different from the Watson-Crick conformation), such as hairpins, cruciform or triplex conformations [24], promoting DNA instability and giving rise to chromosomal reorganizations [25].

While it is clear that tandem repeats are involved in the etiology of several human diseases [26]–[28], the evolutionary implications of these sequences remain elusive. Given that tandem repeats have been shown to be concentrated in evolutionary chromosomal bands in the human genome [10] our aim was to test this hypothesis in other primate species presenting a comprehensive study of the distribution of tandem repeats in great apes. Taking advantage of the sequenced genomes of 10 vertebrate species (chimpanzee, orangutan, rhesus macaque, mouse, rat, horse, dog, cow, opossum and chicken) available in the public databases, we analyzed the distribution of tandem repeats in relation to the distribution of evolutionary breakpoint regions in the human, chimpanzee, orangutan and macaque genomes, from which the ancestral chromosomal state is known. A comparative study among species is presented and its implications for mammalian chromosome evolution are discussed.

## Results

### Whole-genome comparisons and delimitation of homologous synteny blocks (HSBs) and evolutionary breakpoint regions (EBRs) in great apes

#### Definition of HSBs and EBRs

In order to establish the evolutionary genomic landscape in great apes, we initially delimitated HSBs and EBRs in the human, chimpanzee and orangutan genomes by means of pair-wise comparisons (see Material and Methods). The gorilla genome was not available at the moment of the initiation of the study and the rhesus macaque was included as an outgroup for the Hominoidea superfamily.

First, we determined the HSBs and EBRs in the human genome establishing pair-wise whole-genome comparisons with ten vertebrate species (chimpanzee, orangutan, rhesus macaque, mouse, rat, horse, dog, cow, opossum and chicken). The number of HSBs differed depending on the species compared, ranging from 81 HSBs between human and macaque to 470 HSBs between human and opossum (Table 1). HSBs represented more than 70% of the human genome, reaching 91.88% for the human/orangutan comparison (Table 1), reflecting the high conservation of mammalian genomes. The mean length of the HSBs ranged from 30.61 Mbp for human/macaque to 5.08 Mbp for the human/opossum pair-wise comparison. Likewise, the number of EBRs also differed among species, being low in the non-human primate species (35, 61 and 88 between human and macaque, chimpanzee and orangutan, respectively) and high in the human/opossum comparison (Table 1). Moreover, and in order to avoid possible artifacts derived from the low-coverage annotation, intervals longer than 4 Mbp between two HSBs were considered as gaps. Gap regions ranged from 3.79 to 17.82% of the human genome, depending on the genome analyzed (Table 1). The larger percentages of gap regions were found in the human/macaque, human/dog, human/opossum and human/chicken pair-wise comparisons. These differences were probably due to the low coverage of some of the genomes available in the databases (e.g., 5.2X coverage for the macaque genome) or to the large evolutionary distances between species (300 My between human and chicken and 180 My between human and opossum).

**Table 1:** Homologous synteny blocks (HSBs) and evolutionary breakpoint regions (EBRs) in primate genomes.

	HSBs			EBRs			Gaps		
Species compared	N° regions	Total length (Mbp)	% human genome	N° regions	Total length (Mbp)	% human genome	N° regions	Total length (Mbp)	% human genome
**HSA-PTR**	97	2,785	90.86	61	37	1.23	59	138	4.51
**HSA-PPY**	122	2,817	91.88	88	32	1.06	55	116	3.79
**HSA-MMU**	81	2,479	80.86	35	22	0.72	69	460	15.02
**HSA-RNO**	287	2,543	82.95	245	128	4.20	65	289	9.45
**HSA-MMUS**	324	2,727	88.97	291	81	2.64	56	152	4.99
**HSA-ECA**	188	2,764	90.17	154	49	1.62	55	147	4.81
**HSA-BTA**	336	2,726	88.93	301	80	2.62	58	255	8.32
**HSA-CFA**	173	2,388	77.90	128	38	1.24	68	546	17.82
**HSA-MDO**	470	2,390	77.96	424	302	9.86	69	269	8.78
**HSA-GGA**	361	2,176	71.00	288	244	7.97	96	537	17.53
**merged HSA**	1,353	1,403	43.14	898	788	24.24	576	779	23.95
**PTR-HSA**	89	2,832	84.55	32	28	0.85	11	83	2.94
**PPY-HSA**	109	2,888	83.81	46	36	1.04	8	256	7.43
**MMU-HSA**	66	2,466	79.64	27	15	0.48	19	380	12.28

Pair-wise genome comparisons were established in two directions; using as a reference the human genome (HSA-PTR, HSA-PPY, HSA-MMU, HSA-RNO, HSA-MMUS, HSA-ECA, HSA-BTA, HSA-CFA, HSA-MDO, HSA-GGA) or the primate genomes (PTR-HSA, PPY-HSA and MMU-HSA). The total numbers of HSBs, EBRs and gaps in the human genome after merging all pair-wise comparisons are also indicated.

PTR *Pan troglodytes*, PPY *Pongo pygmaeus*, MMU *Macaca mulatta*, RNO *Rattus norvegicus*, MMUS *Mus musculus*, ECA *Equus caballus*, BTA *Bos taurus*, CFA *Canis familiaris*, MDO *Monodelphis domestica* and GGA *Gallus gallus.*

**Figure 1:**
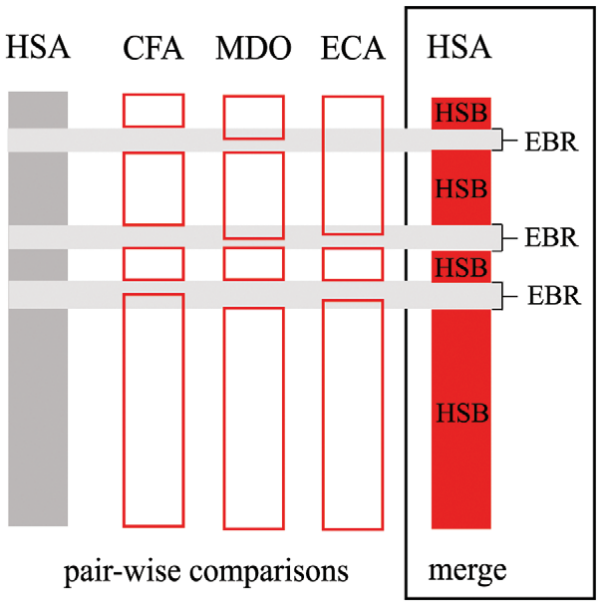
Representation of how homologous synteny blocks (HSBs) and evolutionary breakpoint regions (EBRs) are defined in the human genome. Comparing two genomes at a time, we established pair-wise EBRs. Then, we merged those EBRs that overlap in the same human region, obtaining merged EBRs and HSBs. Abbreviations –PTR: *Pan troglodytes*, ECA: *Equus caballus*, MDO: *Monodelphis domestica*, HSA: *Homo sapiens*.

Given these results, we merged the coordinates of all pair-wise comparisons abovementioned in the human genome (see material and methods for detailed explanation and, Fig. 1) in order to have a broad view of the distribution of evolutionary breakpoint regions. As a result, we obtained a total of 1,353 HSBs and 898 EBRs, representing altogether 67.38% of the whole genome sequence (Table 1). The EBRs detected varied in size, from 3 bp to 3.5 Mbp, with a median length of 304 kbp. Regions of non-coverage (gaps) represented 23.95% of the whole genome whereas telomeric and centromeric regions accounted for the remaining 8.67% (Table 1).

**Figure 2:**
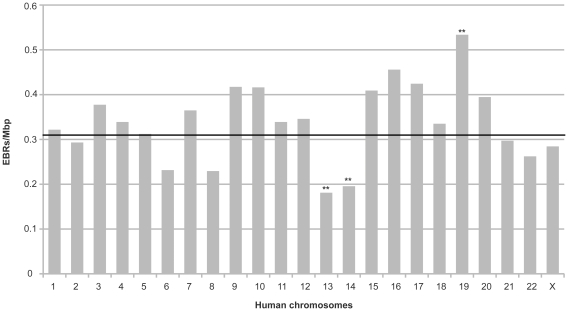
Distribution of EBRs across the human genome. Frequency of EBRs per megabase pair (Mbp) detected on each human chromosome. The dotted line represents the estimated frequency of EBRs per Mbp in the human genome.

We observed that EBRs were unevenly distributed among human chromosomes, given that some human chromosomes accumulated more EBRs than others, independently of their genomic length. We calculated the frequency of EBRs per megabase for each chromosome (Fig. 2), and estimated an average frequency of 0.3 EBR/Mbp in the human genome assuming a homogeneous distribution of the 898 EBRs across the genome (telomeres, centromeres and gap regions were excluded from the analysis). Comparing the observed frequencies with the estimated global frequency of EBRs (0.3 EBRs/Mbp), we observed a deviation (χ^2^ = 7.7, p-value  =  0.005) from the homogeneous distribution of EBRs among chromosomes (Fig. 2). Chromosome 19 accumulated more EBRs (0.53 EBRs/Mbp), while chromosome 13 (0.18 EBRs/Mbp) and chromosome 14 (0.19 EBRs/Mbp) had less EBRs. Although these differences were found to be not significant after Bonferroni correction, the tendency was still observed in all species.

Once the evolutionary regions were defined in the human genome, we determined the HSBs and EBRs in the chimpanzee, orangutan and macaque genomes. In this case, we established pair-wise whole-genome comparisons with the human genome using the primate genomes as references performing chimpanzee/human, orangutan/human and macaque/human pair-wise alignments (Table 1). We detected 32 EBRs in the chimpanzee genome, 46 in orangutan and 27 in macaque, with a median length of 235 kbp, 32 kbp and 10 kbp, respectively. The percentage of homologous syntenic regions was greater in chimpanzee (84.55%) than in orangutan (83.81%) and macaque (79.64%), consistent with their phylogenetic relation to human.

#### Phylogenetic interpretation of evolutionary breakpoint regions

**Figure 3:**
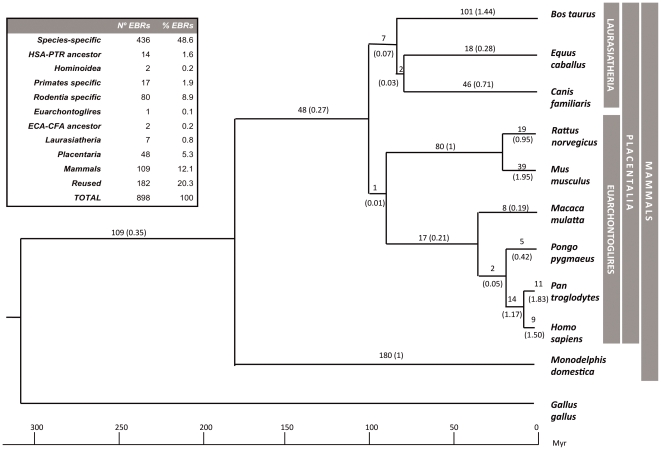
EBRs mapped in the phylogenetic tree of mammalian species included in our study. The phylogeny was based on previous studies [33], [62]. The number of specific evolutionary breakpoint regions detected is plotted in each phylogenetic branch. The number of EBRs per million years detected for each lineage is displayed in brackets. Inset shows the number and percentage of EBRs found in our study.

To have an estimation of the EBR reuse during mammalian evolution we placed the EBRs detected in the human genome in an evolutionary context (Fig. 3). Given that we applied maximum parsimony criteria, these rates represent estimates of change. This approach could led us to ignore the variability due to focusing only on the mapping that requires the fewest genomic changes and to underestimate the true rate of change [29]. Out of the 898 EBRs detected, 436 were species-specific (48.6%), 280 clade-specific (31.2%) and 182 (20.3%) were found in two or more species but not in their common ancestor (reused). Based on the phylogenetic distances described by Murphy and co-workers [30], we estimated an average rate of 0.35 EBRs per million year (myr) for all mammals and 0.27 EBRs/myr for eutherian mammals. Out of the 280 clade-specific EBRs, 180 were marsupialia-specific (1 EBR/myr), 48 were placentalia-specific (0.27 EBR/myr) and 109 were mammalian-specific (0.35 EBR/myr) (Fig. 3). Among the mammalian species studied, the mouse and the rat genome presented the highest estimated rate of genomic changes (1.85 EBRs/myr and 1.95 EBRs/myr, respectively) whereas the macaque was the species with the lowest rate of change (0.2 EBR/myr). Within Laurasiatheria, the cow was the species with the highest rate (1.44 EBR/myr), followed by the dog (0.71 EBR/myr) and the horse (0.28 EBR/myr). Primates showed the lowest estimated rate of change (0.21 EBR/myr), ranging from 0.2 EBR/myr in macaque to 1.83 EBR/myr in chimpanzee.

Taking into account the putative ancestral hominoid karyotype [1], [31] we interpreted the primate-specific EBRs found in each species of great apes. Chromosomes from orangutan, gorilla, chimpanzee and human are highly homologous and only few major reorganizations differentiate their karyotypes [1]. Since their divergence from a common ancestor 14 million years ago (mya) [32], some chromosomal forms have been maintained collinear (chromosomes 6, 13, 19, 20, 21, 22 and X) whereas others suffered inversions and/or lineage-specific fusions. Pair-wise whole-genome comparisons between great apes and human genomes allowed us to refine the number of rearrangements that occurred during hominoid evolution. New insertions were represented by one EBR whereas inversions were caused by two EBRs (Table 2). Regarding collinear chromosomes, we found reorganizations previously undetected in homologous chromosomes 13, 19 and X. In particular, we found a new insertion in orangutan chromosomes 13, X and 19 and a new inversion in chimpanzee chromosome 19. Even though chromosome 8 is collinear in chimpanzee, orangutan and human, we found one EBR due to an insertion in chimpanzee and orangutan but not in human homologous positions. Regarding the reorganized chromosomes, we corroborated the macro reorganizations found in chromosomes 1, 2, 3, 5, 7, 10, 12, 14, 15, 16, 17 and 18 [1], [31]. In chromosome 11, which the orangutan represents the ancestral form, we verified the inversion found in human and chimpanzee, plus an additional EBR in chimpanzee resulted from an insertion of 355 kb. In chimpanzee chromosome 4 we found 3 EBRs, two as a result of the inversion previously described and one from an insertion of 1.5kb. Likewise, an insertion of 227 kb was found in chimpanzee chromosome 9 (Table 2).

**Table 2:** Newly described reorganizations in human (HSA), chimpanzee (PTR) and orangutan (PPY) chromosomes.

Chromosome	HSA	PTR	PPY
**4**	Ancestral	Insertion (121,995,429-121,997,005)	Inversion previously found a
**7**	Inversion previously found a	Inversion (40,154,256-44,613,528)	Ancestral
**8**	Ancestral	Insertion (7,592,222-7,730,288)	Insertion (44,119,443-47,565,927)
**9**	Ancestral	Insertion (42,012,304-42,239,829)	Inversion previously found a
**11**	Inversion previously found b	Insertion (88,294,605-88,650,196)	Ancestral
**13**	Ancestral	Ancestral	Insertion (23,683,269-23,732,315)
**19**	Ancestral	Inversion (41,544,000-42,809,028)	Insertion (24,329,459-27,955,815)
**X**	Ancestral	Ancestral	Insertion (58,752,636-60,465,663)

The ancestral form and type of reorganization with the genomic location are shown. The genomic positions (start and end, NCBI build 36) of each insertion or inversion are also indicated.

a [1],

b [31]

### Tandem repeats analysis

We elaborated a comprehensive study of the distribution of tandem repeats in great apes (macaque, orangutan, chimpanzee and human) with the aim to determine whether there is any correspondence between tandem repeats and the location of evolutionary breakpoint regions in these species.

#### Distribution of tandem repeats

Using the eTandem algorithm, we detected a total of 758,206 tandem repeats in the human genome, grouped into 242,539 different motif types with a repeat unit size ranging from 2bp to 100bp. Similar values were found in macaque, orangutan and chimpanzee: (i) 714,458 tandem repeats representing 229,023 motif types in chimpanzee, (ii) 697,824 tandem repeats grouped into 230,650 motif types in orangutan and (iii) 733,524 tandem repeats corresponding to 211,199 motif types in rhesus macaque. These data suggest that the overall content of tandem repeats in terms of number of tandem repeats is conserved during primate genome evolution. When studied more in detail, we found that the most representative and therefore more frequent motifs were the same in the genomes of all four primate species: CA, AT, AAAT, TC, CAAA and AAAG. These AT-rich tandem repeats accounted for approximately 30% of the whole tandem repeat content in these genomes.

Subsequently, we analyzed the density of tandem repeats in each primate chromosome in order to compare the distribution of tandem repeats among species (File S1). In the human genome, the overall density of tandem repeats varied from 11,682 bp/Mbp in chromosome 14 to 33,091 bp/Mbp in chromosome 19 (File S1). The same pattern was observed in each primate homologous chromosomes. In chimpanzee, the density ranged from 17,253 bp/Mbp (chromosome 14) to 36,446 bp/Mbp (chromosome 19). This pattern is also conserved in orangutan in the homologous chromosome 14, and even in rhesus macaque, where the chromosome 7, homologous to human chromosome 14, has the lowest density (20,460 bp/Mbp).

**Figure 4:**
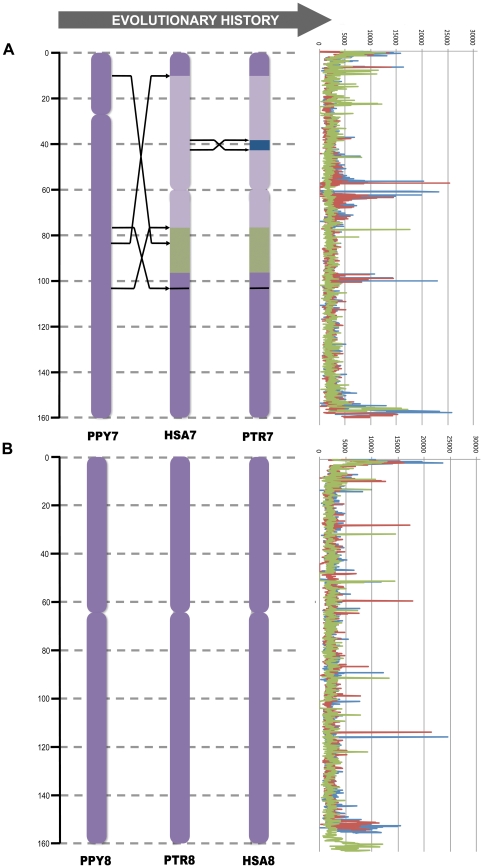
Tandem repeat content (bp) in human chromosomes 8 and 7 and its homologous in chimpanzee, orangutan and macaque. The image represents an example of a reorganized chromosome (a) and a collinear chromosome (b). In each case, the left panel shows the evolutionary history of each chromosome during hominoid evolution. The right panel shows the tandem repeat content in 100 kb windows in human (blue), chimpanzee (red), orangutan (green) and macaque (purple) genomes. Abbreviations –PPY: *Pongo pygmaeus*, PTR: *Pan troglodytes*, HSA: *Homo sapiens*.

Since we observed different tandem repeat density and an uneven distribution of EBRs among primate chromosomes, we decided to analyze thoroughly the tandem repeats landscape of each primate chromosome considering their evolutionary history: which chromosomal form was maintained collinear or suffered any reorganization since their common hominoid ancestor according to previous reports [1], [31]. We scrutinized each chromosome's complete sequence using moving non-overlapping windows of 0.1 Mb in order to analyze the distribution of tandem repeats in each of the primate genomes, using a Kolmogorov-Smirnov test (Fig. 4 and File S2). Those chromosomes that suffered the same evolutionary process seem to have the same tandem repeats distribution while those with different evolutionary history have a statistically different tandem repeats landscape. The tandem repeat distribution of five (PPY6, PTR10, PPY12, HSA18, and PTRX) out of 69 chromosomes analyzed did not correlate with their evolutionary history, suggesting that additional elements are influencing the dynamics of tandem repeats. Herein are the results of the comparison of tandem repeat distributions along each primate chromosome:

#### Chromosome 1

Human chromosome 1 is considered to be the derived form, showing a pericentric inversion when compared to chimpanzee and orangutan chromosome 1. The human tandem repeat landscape also differs from the other two great apes (HSA vs PTR: p-value = 0.006; HSA vs PPY: p-value = 0.000).

#### Chromosome 2

It is well known that human chromosome 2 derives from the ancestral form by a fusion of two hominoid homolog chromosomes [1]. The ancestral 2a form corresponds to HSA2pq and also has suffered a pericentric inversion in the human form, whereas the ancestral 2b form has not suffered further reorganizations. The tandem repeat contour is different between human and the other great apes regarding chromosome 2a form (HSA vs PTR: p-value = 0.000; HSA vs PPY: p-value = 0.000) but is maintained in the homologous chromosome 2b form (HSA vs PTR: p-value = 0.738; HSA vs PPY: p-value = 0.192).

#### Chromosome 3

Human and chimpanzee chromosomes are the derived forms, with an inverted region compared to orangutan chromosome. The tandem repeats distribution confirms this pattern (HSA vs PTR: p-value = 0.062; HSA vs PPY: p-value = 0.009).

#### Chromosome 4

All the great apes have a derivative chromosome 4 that evolved differently since their common ancestor. We found a different tandem repeats distribution between human and chimpanzee forms but the same distribution between human and orangutan forms (HSA vs PTR: p-value = 0.022; HSA vs PPY: p-value = 0.272).

#### Chromosome 5

Human chromosome is considered the ancestral form, whereas the chimpanzee and the orangutan have derived forms due to pericentric inversions. The tandem repeats landscape is consistent with this pattern (HSA vs PTR: p-value = 0.031; HSA vs PPY: p-value = 0.001).

#### Chromosome 6

The three species shared the same chromosome form, which is considered to be ancestral. We found the same tandem repeat profile between human and chimpanzee (HSA vs PTR: p-value = 0.069) but it differs between human and orangutan (HSA vs PPY: p-value = 0.003).

#### Chromosome 7

The orangutan chromosome represents the ancestral form, while human and chimpanzee share a pericentric inversion. We found the same tandem repeats pattern in human and chimpanzee (HSA vs PTR: p-value =  0.203) but this was different in orangutan (HSA vs PPY: p-value = 0.050) (Fig. 4a).

#### Chromosome 8

The three hominoid species share the same form but we detected an insertion of ∼3Mb in the orangutan chromosome 8 (Table 2). This difference is reflected in the tandem repeats landscape, being equal between human and chimpanzee (p-value = 0.128) but different in orangutan (p-value = 0.009) (Fig. 4b).

#### Chromosome 9

All three species have different chromosomal forms, being the orangutan chromosome the ancestral one. Tandem repeats distribution is consistent with these differences (HSA vs PTR: p-value = 0.002; HSA vs PPY: p-value = 0.000).

#### Chromosome 10

Orangutan chromosome 10 is considered to be the ancestral form, which differs from human and chimpanzee forms by a paracentric inversion. We found that human and orangutan have a different tandem repeat pattern (p-value = 0.001) as well as human and chimpanzee (p-value = 0.010), although the same pattern between these two species was expected.

#### Chromosome 11

The ancestral chromosome form is conserved in orangutan, which differs from the human chromosome by a pericentric inversion and from chimpanzee by a pericentric inversion and an insertion of ∼400 Kb (Table 2). These differences are also reflected in the tandem repeat distribution (HSA vs PTR: p-value = 0.016; HSA vs PPY: p-value = 0.000).

#### Chromosome 12

Human and orangutan share the same form, which is considered the ancestral. Chimpanzee differs from them by a pericentric inversion. In this case, the tandem repeats landscape is different between human and chimpanzee (p-value = 0.050) and between human and orangutan (p-value = 0.004).

#### Chromosome 13

Human and chimpanzee share the same form and have the same tandem repeats pattern (p-value = 0.072), while orangutan have a ∼100Kb insertion (Table 2) and shows a different tandem repeats pattern (p-value = 0.003).

#### Chromosome 14

All great apes share the same chromosome form and also the same tandem repeats landscape (HSA vs PTR: p-value = 0.051; HSA vs PPY: p-value = 0.051).

#### Chromosome 15

All great apes have different chromosome forms and different tandem repeats profile (HSA vs PTR: p-value = 0.004; HSA vs PPY: p-value = 0.001).

#### Chromosome 16

All great apes have different chromosome forms and different tandem repeats profile (HSA vs PTR: p-value = 0.001; HSA vs PPY: p-value = 0.000).

#### Chromosome 17

Human and orangutan share the same ancestral form, while chimpanzee suffered a pericentric inversion. This pattern is in agreement with the tandem repeats distribution (HSA vs PTR: p-value = 0.030; HSA vs PPY: p-value = 0.106).

#### Chromosome 18

Chimpanzee and orangutan share a chromosome form ancestral to great apes, which differs from the human by a pericentric inversion. This is not observed in the tandem repeats profile, given that all the species share the same distribution (HSA vs PTR: p-value = 0.095; HSA vs PPY: p-value = 0.206).

#### Chromosome 19, 20, 21 and 22

All great apes share the same chromosome form and also the same tandem repeats landscape [HSA19 (PTR: p-value = 0.127; PPY: p-value = 0.161) HSA20 (PTR: p-value = 0.138; PPY: p-value = 0.051) HSA21 (PTR: p-value = 0.106; PPY: p-value = 0.111) HSA22 (PTR: p-value = 0.082; PPY: p-value = 0.051)].

#### Chromosome X

Human and chimpanzee share the same ancestral form while orangutan has a ∼2Mb insertion (Table 2). Tandem repeat pattern is in agreement with human-orangutan evolution (p-value = 0.021) but not with human-chimpanzee history (p-value = 0.000).

#### Tandem repeats are accumulated in evolutionary breakpoint regions

Once we studied the distribution of tandem repeats across whole genomes, we analyzed whether tandem repeats were differentially accumulated in EBRs and/or HSBs and if this pattern was conserved among species. In all cases, we analyzed two parameters: (i) number of tandem repeat loci, and (ii) number of base pairs implicated in tandem repeats. By this way we took into account not only the number of repeats but also the density of tandem repeats in each genomic region.

We observed 189,330 tandem repeat loci in EBRs and 360,314 loci in HSBs in the human genome. Assuming a homogeneous distribution of tandem repeat loci in these genomic regions, we expected 183,213 and 366,431 tandem repeat loci in EBRs and HSBs, respectively, showing that the observed tandem repeat loci are significantly deviated (p-value < 0.001). Mirroring these results, we also detected that EBRs contained significantly more base pairs implicated in tandem repeats than HSBs in the human genome (contingency analysis, p-value < 0.001).

Therefore, and to have a general overview of the genomic landscape, we used the EBRs and HSBs defined in the human genome to analyze whether there was any specific repeat accumulated in each different genomic region by means of contingency analysis. Out of the 242,539 different motif types found in the human genome, no specific repeat motif was exclusively present in EBRs or HSBs. However, 17 different microsatellite motifs were significantly accumulated in EBRs (p-values ≤ 0.0016) (Table 3). Although we did not detect any pattern regarding the repeat motif and the GC content in the whole tandem repeat content, we found five microsatellites (AAAT, TTTG, TTTC, TATTT and ATTTTT) present in a extremely high frequency in the human genome (more than 1000 repeat units and AT content ≥ 80%) (Table 3). Of these overrepresented tandem repeats, the AAAT motif was by far the most frequent among all EBRs. We, then, analyzed if the distribution of the AAAT motif was dependant on the type of EBRs and we observed an accumulation of this motif in the EBRs not related to primates (p-value < 0.001).

**Table 3:** Microsatellite motifs significantly accumulated in EBRs.

Motif	EBRs		HSBs		p-value
	observed	expected	observed	expected	
aaat*	8186	7373	14336	15148	3.05 E-21
tttg*	3930	3739	7492	7682	0.0018
tttc*	3187	2996	5966	6156	0.0005
tattt*	2488	2328	4624	4783	0.0009
attttt*	1635	1513	2987	3108	0.0017
agg	1011	880	1678	1808	0.000011
agaggg	231	186	339	383	0.0012
ggggga	156	120	212	247	0.0012
tggggg	100	69	111	141	0.0002
cccagc	75	44	61	91	0.000005
gccggg	66	43	68	90	0.0008
ccggc	36	16	15	34	0.000002
ggcagg	36	20	27	42	0.0007
actg	34	19	25	39	0.0008
ggggat	24	11	11	23	0.0002
ctgacc	23	9	5	18	0.000005
ggctct	13	5	4	11	0.0016

Asterisks indicate the overrepresented motifs (more than 1000 repeat units detected, see text for details).

Given the similarity of these microsatellites rich in AT content to the standard L1 cleavage site for classical retrotransposition (5′-TTAAA-3′, [33]), we examined a possible association with any L1 and/or *Alu* sequences in the human genome. In doing so, we considered five possible scenarios: (i) the repeat is not contiguous to any transposable element, (ii) the repeat is upstream or (iii) downstream of the transposable element, (iv) the motif is in-between two transposable elements or (v) two repeat motifs surround one transposable element. Notably, we observed that the AAAT motif was the only repeat significantly associated with *Alu* elements but only when it is located upstream of the transposable element (TE) in EBRs (χ^2^  =  9.33, p-value  =  0.002) but not in HSBs (χ^2^  =  1.99, p-value  =  0.07). Moreover, we found more AAAT motifs associated with *Alu* repeats in primate-specific EBRs than in the other types of EBRs (p-value < 0.001). Regarding the other over-represented repeats (Table 3), none of them was significantly associated with TE elements when EBRs and HSBs were compared (data not-shown). In order to understand the observed association, we analyzed if the distribution of *Alu* sequences was dependant on the type of EBRs (i.e. EBRs primate-specific) given that it is well known that there was a burst of *Alu* transposition in the lineages leading to primates around ∼40 mya [34]. Out of the 1,212,896 *Alu* repeats found in the human genome, 281,019 were located in EBRs. This value represents almost half of the expected number of *Alu* loci assuming a random distribution and shows a depletion of *Alu* sequences in these EBRs (p-value < 0.001). However, when we focused only on the primate-specific EBRs, we found a significant accumulation of *Alu* sequences in these regions (p-value < 0.001). Therefore, our observations indicate that primate-specific EBRs are enriched in *Alu* repeats, but depleted in AAAT motifs when compared to other types of EBRs, although the AAAT motifs found in primate-specific EBRs are significantly associated with *Alu* sequences.

## Discussion

### Homologous synteny and evolutionary breakpoint regions in mammalian genomes

Since the initial whole-genome analysis performed by Murphy and collaborators [8], several studies have described those evolutionary genomic regions involved in the reshuffling of mammalian genomes [6], [7], [10], [11], [35]. Although the focus of these studies was the precise delimitation of the evolutionary breakpoints, the results published to date are far from being consistent. Discrepancies are probably due to differences in the versions of the genomes and the source of the data analyzed (e.g., radiation hybrid maps or whole-genome DNA sequences), differences in the level of resolution of the technique applied, and because the sets of species examined were only partially overlapping. By analyzing the whole-genome sequences of 10 vertebrate species (chimpanzee, rhesus macaque, orangutan, mouse, rat, cow, dog, horse, opossum and chicken) we identified 1,353 vertebrate HSBs (Table 1). This number of homologous synteny blocks is very similar to the previous studies [9], [11], reflecting the high degree of conservation among mammalian genomes. However, we identified substantially fewer EBRs in the human genome (n = 898; median size  =  304 Kb), than previously published [11] probably due to the conservativeness of our approach. Since we excluded centromeric, telomeric and gap regions in our analysis in order to avoid low coverage regions and, therefore, false positives, EBRs and HSBs, represented 67.38% of the human genome. Importantly, when analyzing the distribution of EBRs along the human genome relative to the position in each chromosome we observed a non-homogenous distribution of EBRs among chromosomes (Fig. 2). Specifically, human chromosomes 13 and 14 accumulated fewer and chromosome 19 accumulated more EBR/Mbp than expected. The same pattern was observed in great apes and macaque. Using the non-human primate genomes as a reference we found 32 and 46 EBRs in chimpanzee and orangutan genomes, respectively, non-homogeneously distributed along chromosomes. These results confirm the existence of “hot spot” regions for chromosome evolution supporting the fragile breakage model of chromosome evolution [5]–[11].

Based on chromosomal painting studies, Froenicke [4] established the average rate of chromosomal exchange in eutherian mammals to be 0.19 rearrangements/myr and 0.39 EBR/myr. Combining the data derived from the comparison of 10 mammalian species we estimated a similar rate of evolution (0.35 EBRs/myr).We found that 20.3% of the 898 EBRs detected have been reused during the eutherian evolution. This proportion is higher than the 7-8% described in previous studies [9], [11] but in agreement with initial studies [8]. What it is clear is that a fraction of the mammalian genomes (ranging from 20% to 7%) has suffered recurrent chromosome reorganizations during evolution. We also placed the EBRs detected in an evolutionary context; as an example, we detected 180 EBRs in the lineage leading to the opossum. Since its divergence from the common therian ancestor ∼180 mya, the marsupial species has accumulated a rate of 1 EBR/myr. In placental mammals, the two rodent species studied (mouse and rat) accumulated more clade-specific EBRs (80) than other clades, with a rate of 1 EBR/myr, showing a high rate of EBRs, as previously described in the literature [36]. Primates, on the other hand, show a wide range of rearrangement rates with chimpanzee showing the highest rate of genomic reorganization (1.83 EBRs/myr).

Moreover, and considering the evolutionary history of each hominoid chromosome [1], [31] we were able to refine the rearrangements that occurred during genome evolution in great apes. Among chromosomes that have been conserved since their common ancestor, we found new insertions in orangutan chromosomes 13, 19 and X and an inversion in chimpanzee chromosome 19. In addition, we defined more rearrangements in the reorganized chromosomes. For instance, we found one insertion in chimpanzee chromosome 4, 9 and 11. Even though the great apes genomes are highly conserved, when their sequences are analyzed more in detail, these rearrangements show that they are organized as conserved blocks that had suffered additional reshuffling.

The distribution of EBRs across chromosomes, the high reuse degree of EBRs and the reconstruction of the likely chromosomal architecture of ancestral mammalian genomes have revealed that evolutionary breakpoints are clustered in regions that are prone to disruption, promoting the subsequent reorganization of chromosomes [37], [38]. However, one question remains open: Is there any sequence composition and/or genome organization accounting for the distribution of evolutionary regions? To shed light on this pivotal issue, we have characterized the tandem repeats in the evolutionary regions detected.

### Tandem repeats distribution and its evolutionary implications

We were able to elaborate a tandem repeat database distributed into five different regions (telomeres, centromeres, HSBs, EBRs and gaps) along the genomes of great apes and the macaque. We detected that the overall content of tandem repeats were similar in these closely related species (758,206 tandem repeats in human, 714,458 tandem repeats in chimpanzee, 697,824 in orangutan and 733,524 in the macaque). Moreover, out of the total content of tandem repeats, we observed that six tandem repeat motifs (CA, AT, AAAT, TC, CAAA and AAAG) were highly represented in the primate genomes. The presence of the same six microsatellites in the primate species is somehow surprising despite their common ancestor because microsatellites are highly mutable (in humans: 10^-4^ mutations per locus per generation, [39]). However, this conservation is coherent with the microsatellite turnover theory (i.e. cycles of expansions/deletions and stabilization/reactivation) and suggests that microsatellites fluctuate as a whole [40].

Once we studied the overall content of tandem repeats in the primate genomes, we focused on the distribution of tandem repeats in each chromosome. We observed that not all the chromosomes have the same tandem repeat density (bp implicated in repeats/Mbp of genome) (File S1). The human chromosome 14 and its homologous in the non-human primate species had the lowest density while the human chromosome 19 and its homologous had the highest tandem repeat density. These differences among chromosomes could be due to several factors, such as (i) random amplification and appearance of new repeats, (ii) some selective pressure that restricts the spread of the repeats or, (iii) artifacts of the sequencing procedure itself. Since we have analyzed the tandem repeats distribution in all great ape chromosomes and found the same overall content of tandem repeats, we discard both random amplification and biases in the sequencing procedure. To further analyze these differences, we used sliding windows of 100kb to compare the distribution of tandem repeats in each chromosome of the primate species (Fig. 4 and File S2). We found a non-homogeneous distribution of tandem repeats, with a high accumulation in the pericentromeric and telomeric regions, mirroring previous results [10]. But, more importantly, we found differences in tandem repeat distributions among species, suggesting that they might be correlated with the evolutionary history of each primate chromosome. Roughly, our qualitative comparisons of chromosome evolution suggest that the tandem repeats landscape might have been conserved in collinear chromosomes, but altered in those reorganized chromosomes (Fig. 4 and File S2). Further analysis will be necessary in order to corroborate this hypothesis.

The analysis of the human genome revealed specific features not found in the other primate species analyzed. Excluding regions of high complexity from our analyses (telomeres, centromeres and gaps) EBRs in the human genome accumulated more tandem repeat base-pairs than HSBs (p≤0.05 and p≤0.001). This result confirms previous observations [10] indicating that tandem repeats are elements that could promote genome reorganization during the evolutionary process. With the aim to investigate whether there was any particular DNA configuration or composition driving genome instability we analyzed more in detail the distribution of tandem repeats across the human genome. Although no specific repeat motif was exclusively present in EBRs or HSBs, 17 different microsatellites motifs were significantly accumulated in EBRs. Notably, out of these overrepresented tandem repeats, the AAAT was the most frequently detected. It has been described that this motif could form single-stranded coils [24], favoring chromatin instability and increasing the likelihood to break.

Additionally, the observed association of some tandem repeats with L1 and *Alu* elements provides indications for the possible role of transposable elements in shaping the distribution of mammalian large-scale chromosomal changes. Transposable elements, such as *Alu* and LINEs, are well known to induce genomic reorganizations and structural variation through multiple pathways, including unequal homologous recombination and alternative transposition, for instance [20], [41]–[44,]. Although the association between microsatellites and transposable elements has been previously reported [45], [46], the origin of this association remains unclear. The association of AAAT and transposable elements found in our study can be explained by (at least) two non-mutually exclusive hypotheses. One possibility is that the presence of the AAAT microsatellite in certain regions could derive from transposable elements already inserted in the genome. This interpretation is plausible, since both L1 and *Alu* are characterized by a 3′ poly(dA)-rich tail and an internal tandem repeat region [47]. Furthermore, Abrusan and Krambeck [48] have described that these transposable elements are enriched in AT-rich regions in the human genome. Alternatively, AAAT repeats could represent likely target regions for L1 and/or *Alu* insertions since this motif closely resembles the canonical cleavage sites for these elements (5′-TTAAA-3′) [33]. Cleavage on both strands is required, resulting in an intermediate equivalent to double-strand breaks (DSBs) at the early stage of the reverse transcription reaction. Gasior and collaborators [49] demonstrated an excess of DSBs in the L1 transposition process. The reasons for this high quantity of DSBs are unknown. Although the host cell would successfully repair most of the DSBs created, a fraction of these can be misrepaired, and eventually induce chromosomal alterations [49].

As a preliminary survey to favor one of these two hypotheses, we analyzed the distribution of *Alu* sequences and AAAT motifs in the different types of EBRs. We observed an accumulation of *Alu* repeats and depletion in number of AAAT motifs in primate-specific EBRs when compared with the other types of EBRs. Considering the massive transposition of *Alu* sequences that occurred in the lineages leading to primates around ∼40 mya [34] a very appealing scenario is to consider the AAAT motif as the target site of insertion of *Alu* sequences. Under such scenario, the enrichment of AAAT-*Alu* association observed in primate-specific EBRs could represent signatures of ancient insertions. Favoring this hypothesis, it has been previously shown that *Alu* density strongly correlates with L1 target site insertion motif and regions more prone to DSBs formation [50]. At this point, however, a detailed pair-wise comparison between closely related species in which the ancestral state of a novel insertion can be identified would allow us to distinguish if the accumulation of AAAT motifs is the cause or consequence of L1 and *Alu* insertions.

Summarizing, our results provide evidences for the role of both tandem repeats and transposable elements in evolution. A plausible hypothesis is to consider that an accumulation of tandem repeats in certain genomic regions might form secondary structures in the DNA and, therefore, promotes genome instability that could lead to evolutionary chromosomal changes. Moreover, certain tandem repeats (i.e. AAAT) could work as target sites, promoting the insertion of transposable elements and, eventually, leading to genomic reorganizations by non-allelic homologous recombination (NAHR) [43]. Previous studies have reported how breakpoint regions are rich in segmental duplications [15]–[17], [51], [52], high repeat content [10], [14], transposable elements [19], [20], [53], [54] or long regulatory regions. This heterogeneity in results is suggesting that additional elements, not only the DNA sequence per se, are affecting breakage susceptibility. Recent data indicate that the permissiveness of some regions of the genome to undergo chromosomal breakage could be determined by changes in chromatin conformation [19], [32]. In this sense, transposable elements have been reported to be associated with the epigenetic status of the genome and regulation of gene expression [55], [56], but also the length and type of tandem repeats can determine the conformation of the chromatin [57]. Although at this point a cause/effect between tandem repeats and genomic instability cannot be determined, we can anticipate, as a working hypothesis, that certain properties of local DNA sequences such as repetitive elements related to open chromatin configurations can be involved in the origin/resolution of chromosomal reorganizations.

## Materials and Methods

### Definition of evolutionary breakpoint regions

We included in our analysis the whole-genome sequences of 10 vertebrate species available in the public databases (Ensembl [58]). These species were chosen based on the availability of their completed whole-genome sequences and they included: *Pan troglodytes* (CHIMP2.1, assembly of March, 2006), *Macaca mulatta* (Mmul_1, assembly of February, 2006), *Pongo pygmaeus* (PPYG2, assembly of April, 2007), *Mus musculus* (NCBIm37, assembly of April, 2007), *Rattus norvegicus* (RGSC 3.4, assembly of December, 2004), *Bos taurus* (Btau_4.0, assembly of October, 2007), *Canis familiaris* (CanFam 2.0, assembly of May, 2005), *Equus caballus* (EquCab2, assembly of September, 2007), *Monodelphis domestica* (MonDom5, assembly of October, 2006) and *Gallus gallus* (WASHUC2, assembly of May, 2006). In addition, we used the human genome (NBCI build 36, assembly of March, 2006) as a reference.

We first defined the homologous synteny blocks (HSBs) and the evolutionary breakpoint regions (EBRs) in the human genome. To do so, we downloaded the pair-wise whole-genome comparisons detailed in the Ensembl genome browser (release 52) between the human reference genome and those of other vertebrate species (chimpanzee, orangutan, macaque, mouse, rat, horse, cattle, dog, opossum and chicken). These pair-wise comparisons were based on sequence homology. For each pair-wise comparison between the human genome and any of the vertebrate species, we established homologous syntenic regions, defining the start and end positions according the Ensembl database (in pb) (Fig. 1). Then, we manually grouped together those syntenic regions spaced less than 4 Mbp, with the same orientation and located in the same chromosome to form a single HSB. In order to avoid possible artifacts derived from the low-coverage annotation of whole-genome sequences, intervals between two contiguous HSBs larger than 4 Mbp in size were considered to be “gaps”. Subsequently, we merged the coordinates of all pair-wise comparisons in the human genome by means of Perl scripts in order to define the total number, position and length of HSBs and EBRs in reference genome (Fig. 1). EBRs were considered as the interval between two contiguous HSBs as described in Ruiz-Herrera and collaborators [10] and were defined by sequence coordinates in any of the nine mammalian species compared with human plus the chicken (Fig. 1). We calculated the percentage of coverage of each pair-wise comparison using the human genome total length (data in Mbp) excluding the Y chromosome from the analysis. Furthermore, we labeled as telomeric/subtelomeric the 2 Mbp at the ends of each human chromosome and as centromeric/pericentromeric the 2 Mbp regions flanking the unknown nucleotides (Ns) as described elsewhere [10]. Thus, the human reference genome was classified into 5 types of genomic regions (telomeres, centromeres, HSBs, EBRs and gaps) in order to proceed with the subsequent analysis.

The definition of homologous synteny blocks (HSBs) and evolutionary breakpoint regions (EBRs) for the primate genomes (chimpanzee, orangutan and macaque) was done following the same approach as with the human genome. In all cases, we wrote Perl scripts to parse the pair-wise comparisons data and to cross-reference the coordinates of all types of genomic regions.

### Phylogenetic interpretation of evolutionary breakpoint regions

Extant mammals are classified into three major groups: monotremes, marsupials and placental mammals (eutherians) that split off from their last shared common ancestor nearly 240 mya [59]. Among placental mammals, four superordinal clades (Afrotheria, Xenarthra, Laurasiatheria and Euarchontoglires/Supraprimates) are recognized based on the phylogenetic analysis of both nuclear and mitochondrial DNA [60]. We followed the phylogeny proposed by Murphy et al. [30] for our phylogenetic interpretations. We classified all EBRs into different types depending on which species they have occurred in: i) species-specific, ii) clade-specific, when the EBR is found in species of the same order or superorder and iii) reused, if the EBR is found in two taxa but not in their common ancestor. We used the maximum parsimony criterion to place events in the tree and obtained the rate estimates in each branch.

### Tandem repeat analysis

We analyzed the distribution of tandem repeats in the human genome using the eTandem algorithm (part of EMBOSS 6.0.1 package [61]). We run the eTandem algorithm with a minimum repeat unit of 2 bp and a maximum repeat unit of 100 bp. The resulting output files were computed for the detection of overlapping tandem repeats and the canonical motif was reported for each repeat (a canonical motif is intended as all possible rotations and reverse complementation; e.g., AC is the canonical form of AC, CA, GT, and TG). We merged the positions of all the canonical motifs detected with those of the different types of genomic regions described in the previous section.

For the analysis of human retroelements, we obtained the genomic positions of all the human L1 and *Alu* sequences described in the UCSC database (http://genome.ucsc.edu) in order to analyze if tandem repeats were immediately contiguous to any transposable element sequence. First, we analyzed if a given motif was associated with L1 or *Alu* sequences within EBRs or HSBs designing a Perl script to compare the positions of the tandem repeats and the transposable elements. By means of a *χ^2^* test we evaluated this association, using the total number of tandem repeats contiguous to L1 and/or *Alu* sequences as the sample and the position in EBRs or HSBs as the factor. In order to analyze the distribution of *Alu* repeats in the different type of EBRs, we applied a *χ^2^* test and we calculated the expected *Alu* loci in each genomic region assuming a homogeneous distribution.

Using Perl scripts, we computed the overlapping degree of tandem repeats, searched the canonical motifs, and merged the positions of tandem repeats with the different types of genomic regions the human genome was classified and with the transposable elements.

### Statistical analyses

We performed the statistical analyses using the JMP 7 package. Centromeric, telomeric and gap regions were excluded before any statistical analyses were performed given that they represent regions of high complexity overall.

To assess if EBRs were evenly distributed across human chromosomes we estimated an average frequency of 0.3 EBR/Mbp assuming a homogeneous distribution of EBRs across the human genome. We used a *χ^2^* test with a Bonferroni correction (p-value  = 0.0022) to evaluate any possible deviation.

For the analysis of tandem repeat distribution, we first compared whether EBRs accumulate more base-pairs involved in tandem repeats than HSBs using a *χ^2^* test. We also analyzed the tandem repeat loci in EBRs and HSBs using the same test. We computed the expected number of tandem repeats in each region by assuming a homogeneous distribution of the total tandem repeat loci along the genome and then distributed them proportionally to the length of each genomic region (EBRs or HSBs). Then, we used a *χ^2^* test with the Bonferroni correction to assess whether a tandem repeat motif accumulates significantly in a certain type of genomic region (p-value =  0.0017).

Finally, to compare the tandem repeat distribution along primate chromosomes we counted the base-pairs of tandem repeats in 100 kb windows for each chromosome. In order to analyze whether the primate genomes had the same tandem repeat landscape, we performed Kolmogorov-Smirnov tests by pairs, comparing all hominoids' chromosomes. P-values smaller than 0.005 indicated that the distribution of base-pairs implicated in tandem repeats were significantly different among species.

## Supporting Information

File S1 **Density of tandem repeats in each primate chromosomes.** The density is expressed in base-pairs (bp) of a tandem repeat sequence per megabase-pairs (Mbp) of a chromosome sequence.
(PDF)

File S2 **Tandem repeat content (bp) in non-overlapping 100 kb windows.** For each chromosome**,** the tandem repeat distribution for human (black), chimpanzee (dark green), orangutan (light green) and macaque (orange) is shown. In each case, the Spearman's p test comparing chimpanzee (PTR), orangutan (PPY) and macaque (MMU) with human (HSA) is indicated. C: centromere, N: distal telomere. * Statistically significant p-value <0.0001.
(PDF)

## References

[1] Yunis JJ, Prakash O (1982). The origin of man: a chromosomal pictorial legacy.. Science.

[2] Clemente IC, Ponsa M, Garcia M, Egozcue J (1990). Evolution of the Simiiformes and the phylogeny of human chromosomes.. Hum.Genet.

[3] Wienberg J, Stanyon R (1997). Comparative painting of mammalian chromosomes.. Curr.Opin.Genet.Dev.

[4] Froenicke L (2005). Origins of primate chromosomes - as delineated by Zoo-FISH and alignments of human and mouse draft genome sequences.. Cytogenet.Genome Res.

[5] Ruiz-Herrera A, Garcia F, Mora L, Egozcue J, Ponsa M (2005). Evolutionary conserved chromosomal segments in the human karyotype are bounded by unstable chromosome bands.. Cytogenet.Genome Res.

[6] Bourque G, Pevzner PA, Tesler G (2004). Reconstructing the genomic architecture of ancestral mammals: lessons from human, mouse, and rat genomes.. Genome Res.

[7] Zhao S, Shetty J, Hou L, Delcher A, Zhu B (2004). Human, mouse, and rat genome large-scale rearrangements: stability versus speciation.. Genome Res.

[8] Murphy WJ, Larkin DM, Everts-van der Wind A, Bourque G, Tesler G (2005). Dynamics of mammalian chromosome evolution inferred from multispecies comparative maps.. Science.

[9] Ma J, Zhang L, Suh BB, Raney BJ, Burhans RC (2006). Reconstructing contiguous regions of an ancestral genome.. Genome Res.

[10] Ruiz-Herrera A, Castresana J, Robinson TJ (2006). Is mammalian chromosomal evolution driven by regions of genome fragility?. Genome Biol.

[11] Larkin DM, Pape G, Donthu R, Auvil L, Welge M (2009). Breakpoint regions and homologous synteny blocks in chromosomes have different evolutionary histories.. Genome Res.

[12] Pevzner P, Tesler G (2003). Human and mouse genomic sequences reveal extensive breakpoint reuse in mammalian evolution.. Proc.Natl.Acad.Sci.U.S.A.

[13] Sankoff D (2009). The where and wherefore of evolutionary breakpoints.. J.Biol..

[14] Kehrer-Sawatzki H, Szamalek JM, Tanzer S, Platzer M, Hameister H (2005). Molecular characterization of the pericentric inversion of chimpanzee chromosome 11 homologous to human chromosome 9.. Genomics.

[15] Bailey JA, Eichler EE (2006). Primate segmental duplications: crucibles of evolution, diversity and disease.. Nat.Rev.Genet.

[16] Kehrer-Sawatzki H, Cooper DN (2008). Molecular mechanisms of chromosomal rearrangement during primate evolution.. Chromosome Res.

[17] Carbone L, Vessere GM, ten Hallers BF, Zhu B, Osoegawa K (2006). A high-resolution map of synteny disruptions in gibbon and human genomes.. PLoS Genet.

[18] Caceres M, Ranz JM, Barbadilla A, Long M, Ruiz A (1999). Generation of a widespread Drosophila inversion by a transposable element.. Science.

[19] Carbone L, Harris RA, Vessere GM, Mootnick AR, Humphray S (2009). Evolutionary breakpoints in the gibbon suggest association between cytosine methylation and karyotype evolution.. PLoS Genet.

[20] Longo MS, Carone DM, Green ED, O'Neill MJ, NISC Comparative Sequencing Program (2009). Distinct retroelement classes define evolutionary breakpoints demarcating sites of evolutionary novelty.. BMC Genomics.

[21] Lander ES, Linton LM, Birren B, Nusbaum C, Zody MC (2001). Initial sequencing and analysis of the human genome.. Nature.

[22] Naslund K, Saetre P, von Salome J, Bergstrom TF, Jareborg N (2005). Genome-wide prediction of human VNTRs.. Genomics.

[23] Armour JA (2006). Tandemly repeated DNA: why should anyone care?. Mutat.Res.

[24] Bacolla A, Larson JE, Collins JR, Li J, Milosavljevic A (2008). Abundance and length of simple repeats in vertebrate genomes are determined by their structural properties.. Genome Res.

[25] Kolb J, Chuzhanova N, Hogel J, Vasquez KM, Cooper DN (2009). Cruciform-forming inverted repeats appear to have mediated many of the microinversions that distinguish the human and chimpanzee genomes.. Chromosome Res.

[26] Knight SJ, Flannery AV, Hirst MC, Campbell L, Christodoulou Z (1993). Trinucleotide repeat amplification and hypermethylation of a CpG island in FRAXE mental retardation.. Cell.

[27] Campuzano V, Montermini L, Molto MD, Pianese L, Cossee M (1996). Friedreich's ataxia: autosomal recessive disease caused by an intronic GAA triplet repeat expansion.. Science.

[28] Usdin K, Grabczyk E (2000). DNA repeat expansions and human disease.. Cell Mol.Life Sci.

[29] Nielsen R (2002). Mapping mutations on phylogenies.. Syst.Biol.

[30] Murphy WJ, Eizirik E, Johnson WE, Zhang YP, Ryder OA (2001). Molecular phylogenetics and the origins of placental mammals.. Nature.

[31] Muller S, Wienberg J (2001). “Bar-coding” primate chromosomes: molecular cytogenetic screening for the ancestral hominoid karyotype.. Hum.Genet.

[32] Goodman M, Porter CA, Czelusniak J, Page SL, Schneider H (1998). Toward a phylogenetic classification of Primates based on DNA evidence complemented by fossil evidence.. Mol.Phylogenet.Evol.

[33] Jurka J (1997). Sequence patterns indicate an enzymatic involvement in integration of mammalian retroposons.. Proc.Natl.Acad.Sci.U.S.A.

[34] Shen MR, Batzer MA, Deininger PL (1991). Evolution of the master Alu gene(s).. J.Mol.Evol.

[35] Lemaitre C, Zaghloul L, Sagot MF, Gautier C, Arneodo A (2009). Analysis of fine-scale mammalian evolutionary breakpoints provides new insight into their relation to genome organisation.. BMC Genomics.

[36] Graphodatsky AS, Yang F, Dobigny G, Romanenko SA, Biltueva LS (2008). Tracking genome organization in rodents by Zoo-FISH.. Chromosome Res.

[37] Froenicke L, Caldes MG, Graphodatsky A, Muller S, Lyons LA (2006). Are molecular cytogenetics and bioinformatics suggesting diverging models of ancestral mammalian genomes?. Genome Res.

[38] Robinson TJ, Ruiz-Herrera A (2008). Defining the ancestral eutherian karyotype: a cladistic interpretation of chromosome painting and genome sequence assembly data.. Chromosome Res.

[39] Ellegren H (2004). Microsatellites: simple sequences with complex evolution.. Nat. Rev.Genet.

[40] Buschiazzo E, Gemmell NJ (2006). The rise, fall and renaissance of microsatellites in eukaryotic genomes.. Bioessays.

[41] Gray YH (2000). It takes two transposons to tango: transposable-element-mediated chromosomal rearrangements.. Trends Genet.

[42] Ostertag EM, Kazazian HH (2001). Twin priming: a proposed mechanism for the creation of inversions in L1 retrotransposition.. Genome Res.

[43] Lee J, Han K, Meyer TJ, Kim HS, Batzer MA (2008). Chromosomal inversions between human and chimpanzee lineages caused by retrotransposons.. PLoS One.

[44] Cordaux R, Batzer MA (2009). The impact of retrotransposons on human genome evolution.. Nat.Rev.Genet.

[45] Nadir E, Margalit H, Gallily T, Ben-Sasson SA (1996). Microsatellite spreading in the human genome: evolutionary mechanisms and structural implications.. Proc.Natl.Acad.Sci.U.S.A.

[46] Kelkar YD, Tyekucheva S, Chiaromonte F, Makova KD (2008). The genome-wide determinants of human and chimpanzee microsatellite evolution.. Genome Res.

[47] Babushok DV, Kazazian HH (2007). Progress in understanding the biology of the human mutagen LINE-1.. Hum.Mutat.

[48] Abrusan G, Krambeck HJ (2006). The distribution of L1 and Alu retroelements in relation to GC content on human sex chromosomes is consistent with the ectopic recombination model.. J.Mol.Evol.

[49] Gasior SL, Wakeman TP, Xu B, Deininger PL (2006). The human LINE-1 retrotransposon creates DNA double-strand breaks.. J.Mol.Biol.

[50] Kvikstad EM, Makova KD (2010). The (r)evolution of SINE versus LINE distributions in primate genomes: sex chromosomes are important.. Genome Res.

[51] Goidts V, Szamalek JM, Hameister H, Kehrer-Sawatzki H (2004). Segmental duplication associated with the human-specific inversion of chromosome 18: a further example of the impact of segmental duplications on karyotype and genome evolution in primates.. Hum.Genet.

[52] Zhao H, Bourque G (2009). Recovering genome rearrangements in the mammalian phylogeny.. Genome Res.

[53] Bourque G Transposable elements in gene regulation and in the evolution of vertebrate genomes.. Curr.Opin.Genet.Dev.

[54] Delprat A, Negre B, Puig M, Ruiz A (2009). The transposon Galileo generates natural chromosomal inversions in Drosophila by ectopic recombination.. PLoS One.

[55] Hua-Van A, Le Rouzic A, Boutin TS, Filee J, Capy P (2011). The struggle for life of the genome's selfish architects.. Biol.Direct.

[56] Slotkin RK, Martienssen R (2007). Transposable elements and the epigenetic regulation of the genome.. Nat.Rev.Genet.

[57] Vinces MD, Legendre M, Caldara M, Hagihara M, Verstrepen KJ (2009). Unstable tandem repeats in promoters confer transcriptional evolvability.. Science.

[58] Hubbard TJ, Aken BL, Beal K, Ballester B, Caccamo M (2007). Ensembl 2007.. Nucleic Acids Res.

[59] Springer MS, Murphy WJ, Eizirik E, O'Brien SJ (2003). Placental mammal diversification and the Cretaceous-Tertiary boundary.. Proc.Natl.Acad.Sci.U.S.A.

[60] Nikolaev S, Montoya-Burgos JI, Margulies EH, Rougemont J, NISC Comparative Sequencing Program (2007). Early history of mammals is elucidated with the ENCODE multiple species sequencing data.. PLoS Genet.

[61] Rice P, Longden I, Bleasby A (2000). EMBOSS: the European Molecular Biology Open Software Suite.. Trends Genet.

[62] Nakatani Y, Takeda H, Kohara Y, Morishita S (2007). Reconstruction of the vertebrate ancestral genome reveals dynamic genome reorganization in early vertebrates.. Genome Res.

