# Supplementary figures and images for: Assessing the Role of Tandem Repeats in Shaping the Genomic Architecture of Great Apes
Source: PLoS One. 2011 Nov 4;6(11):e27239. doi: 10.1371/journal.pone.0027239 (PMC3208591; doi:10.1371/journal.pone.0027239)

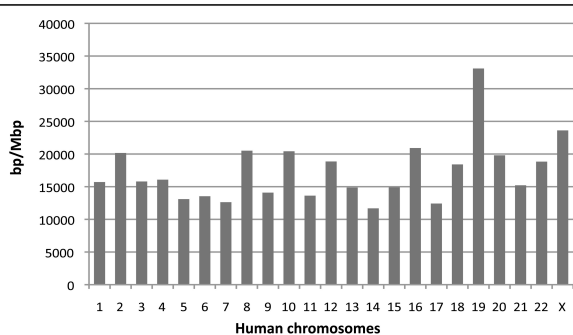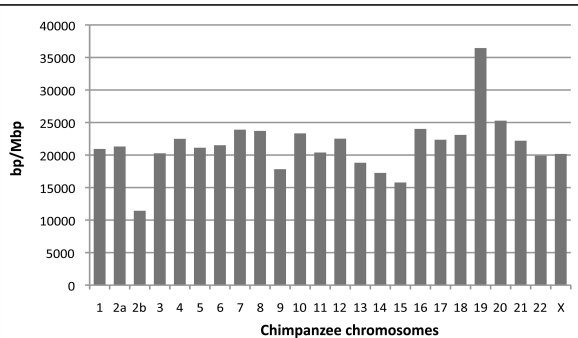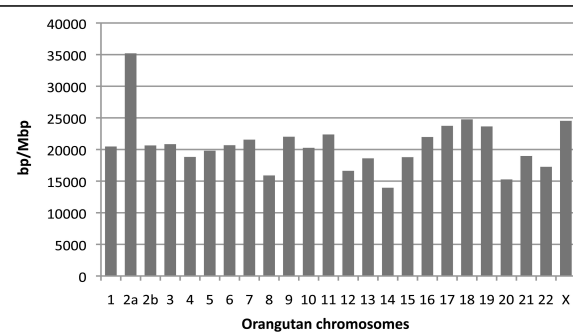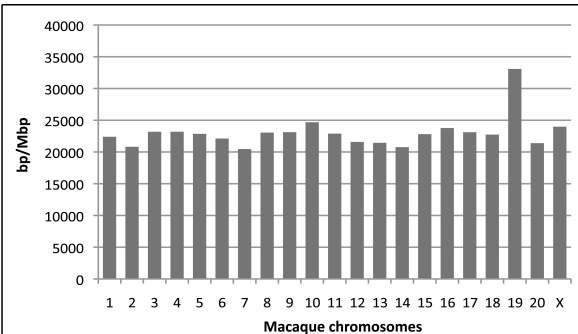

Supplement: File S1 — Density of tandem repeats in each primate chromosomes. The density is expressed in base-pairs (bp) of a tandem repeat sequence per megabase-pairs (Mbp) of a chromosome sequence. (PDF) [file pone.0027239.s001.pdf]

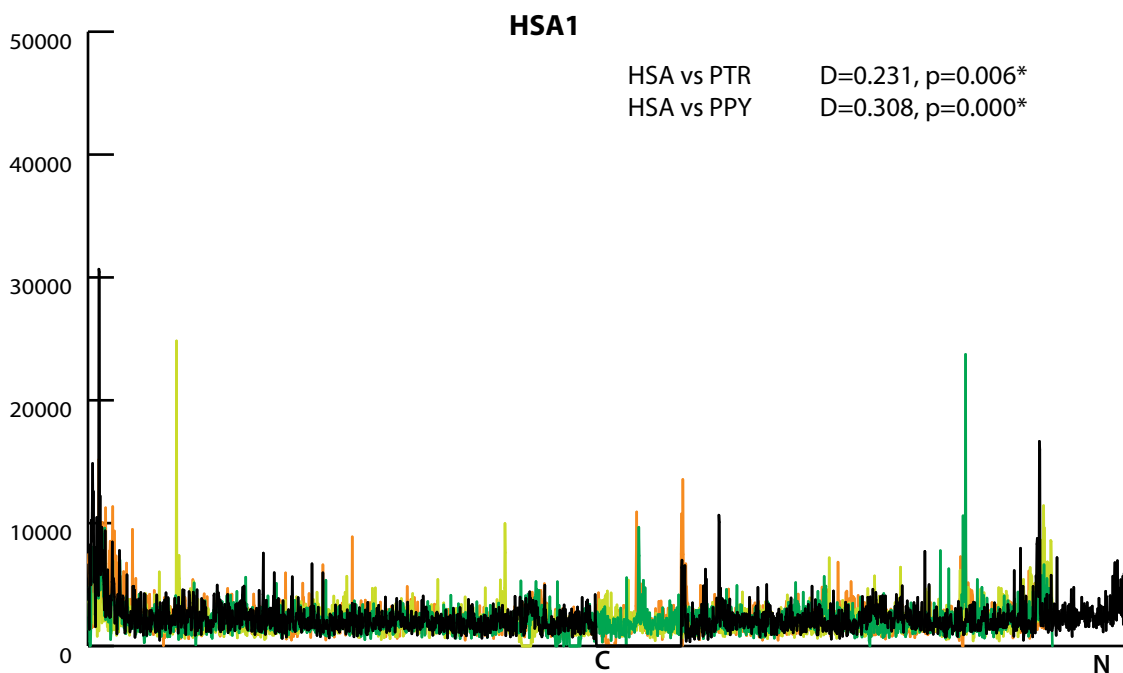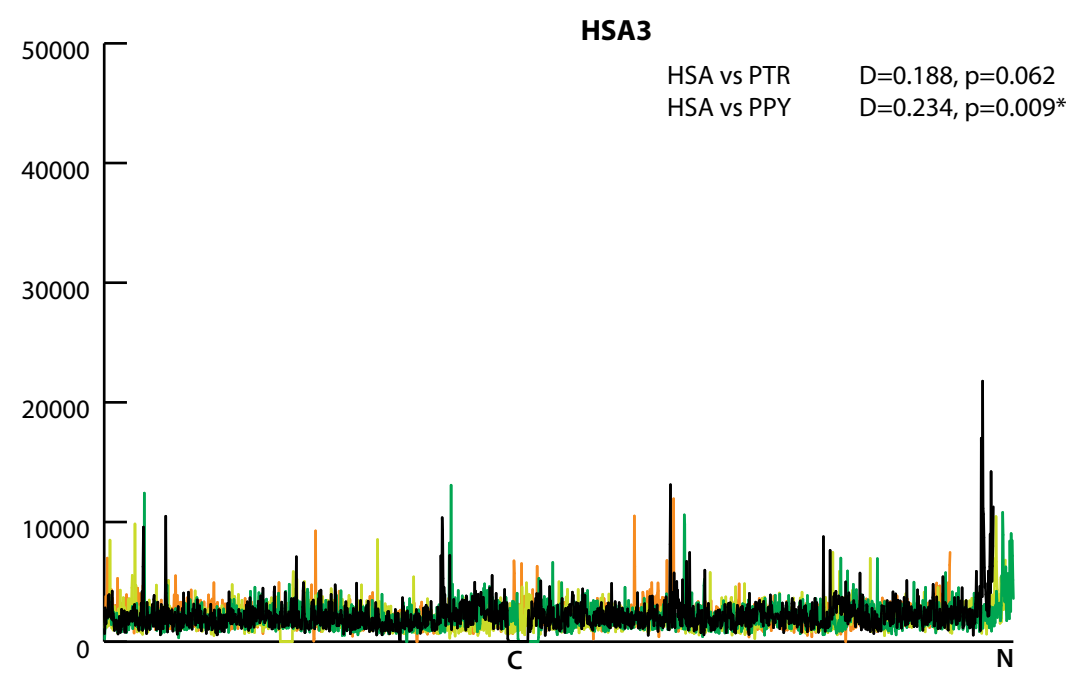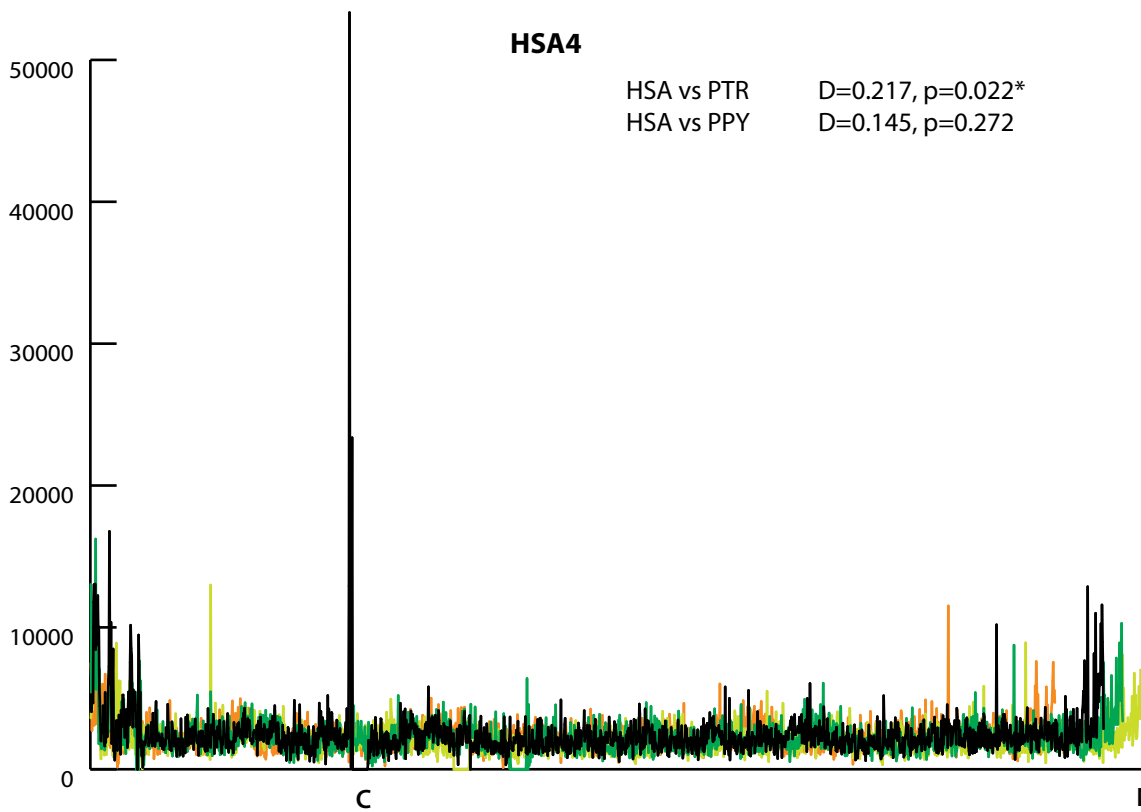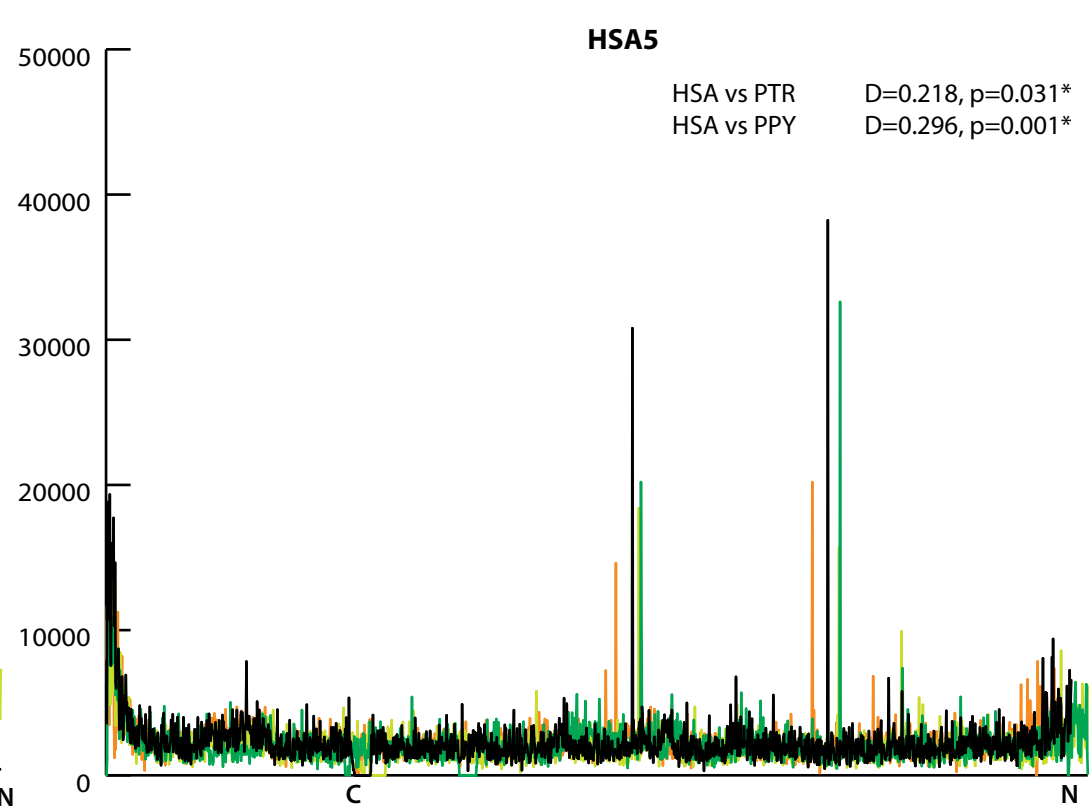

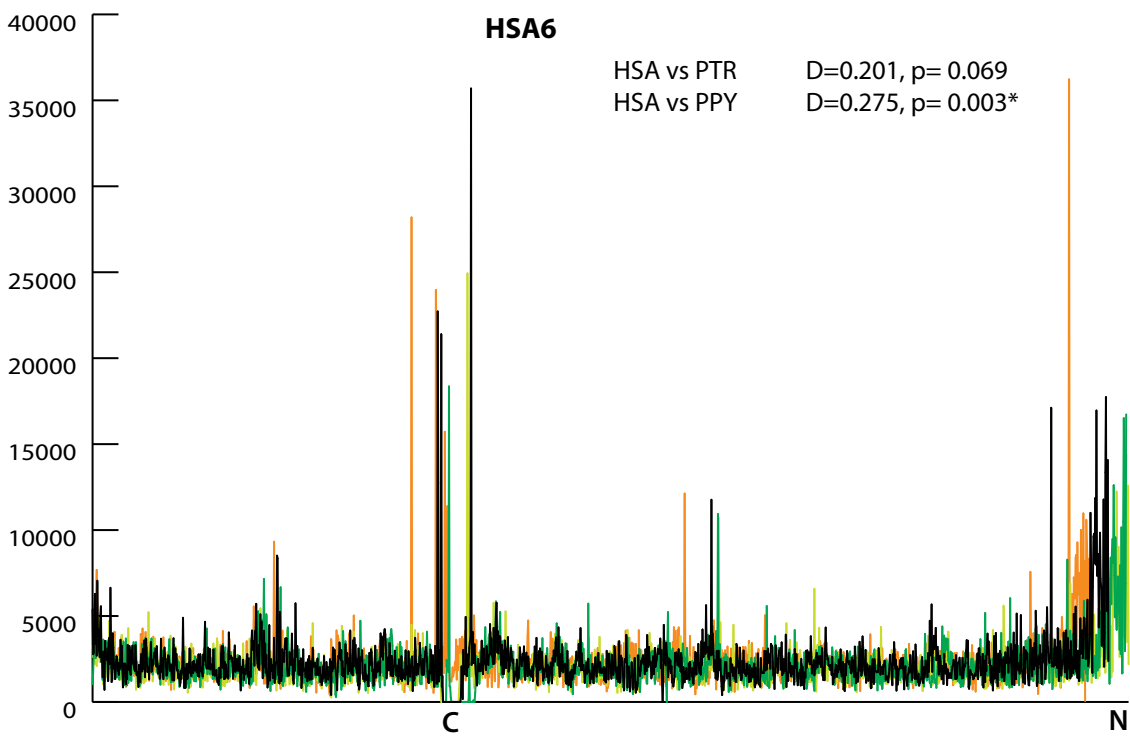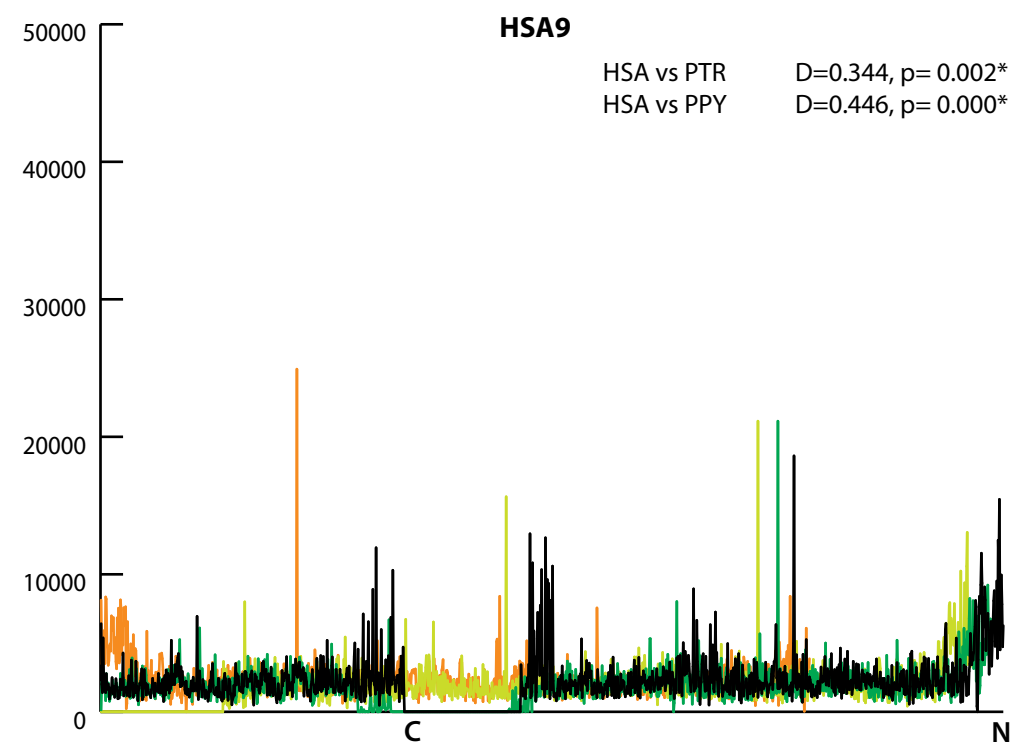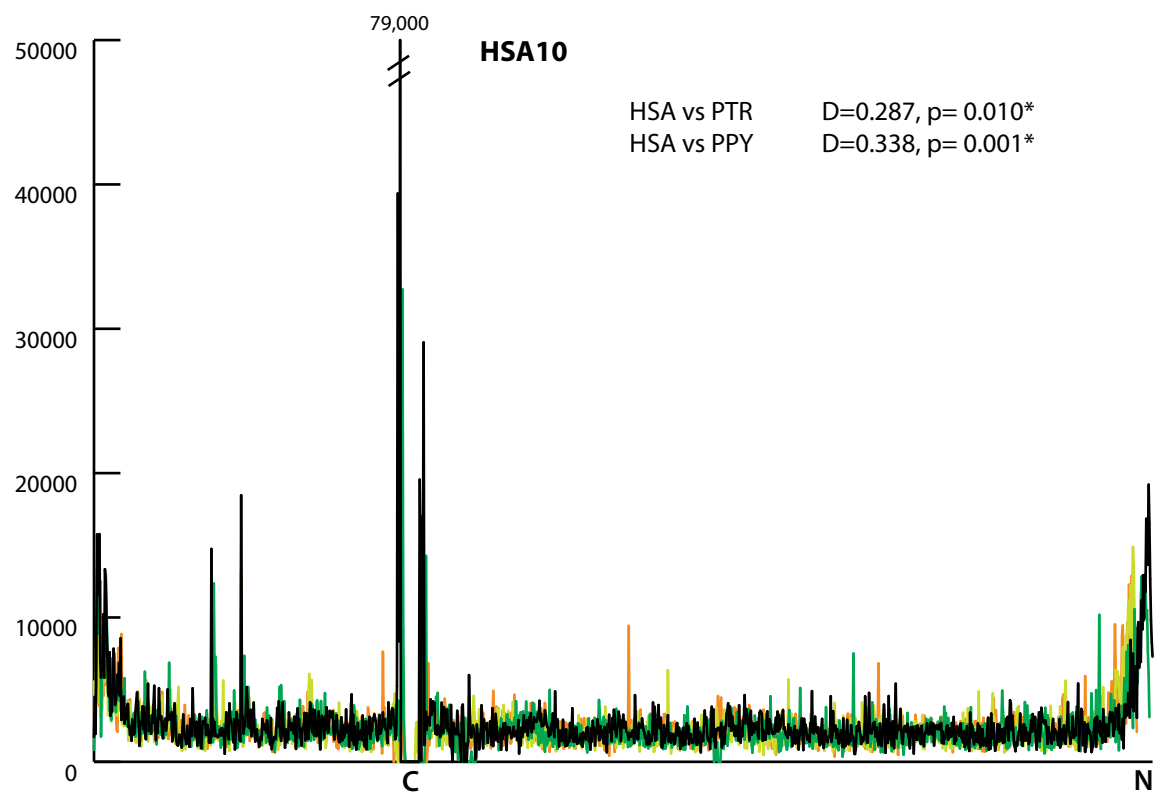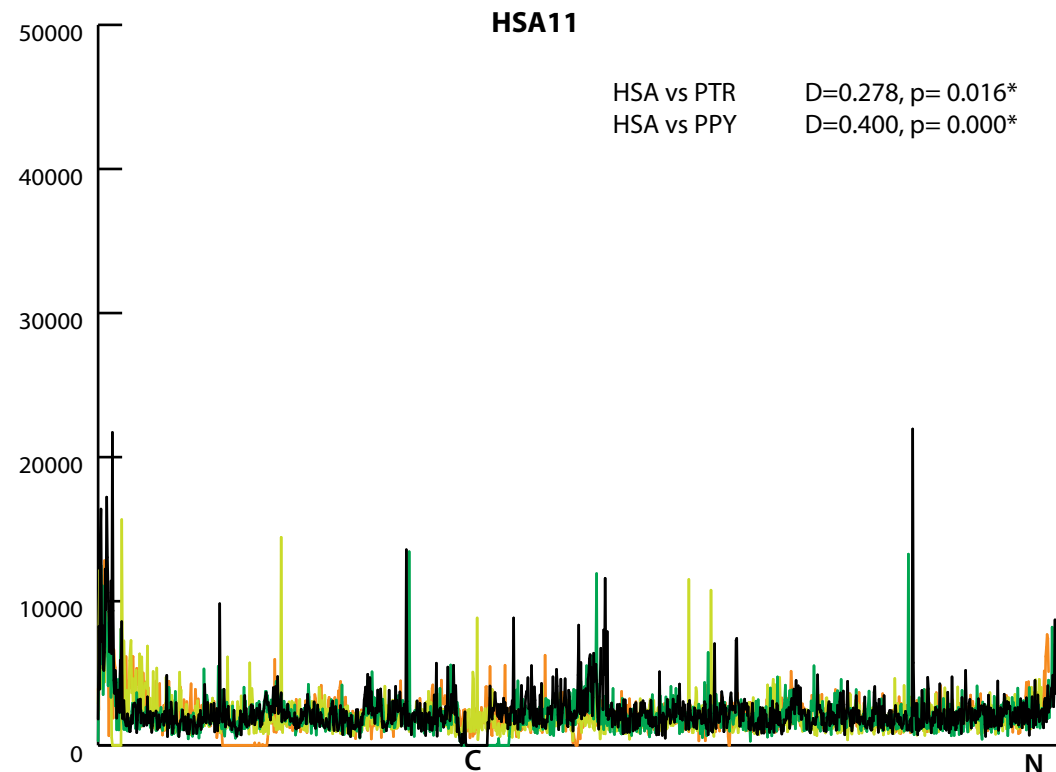

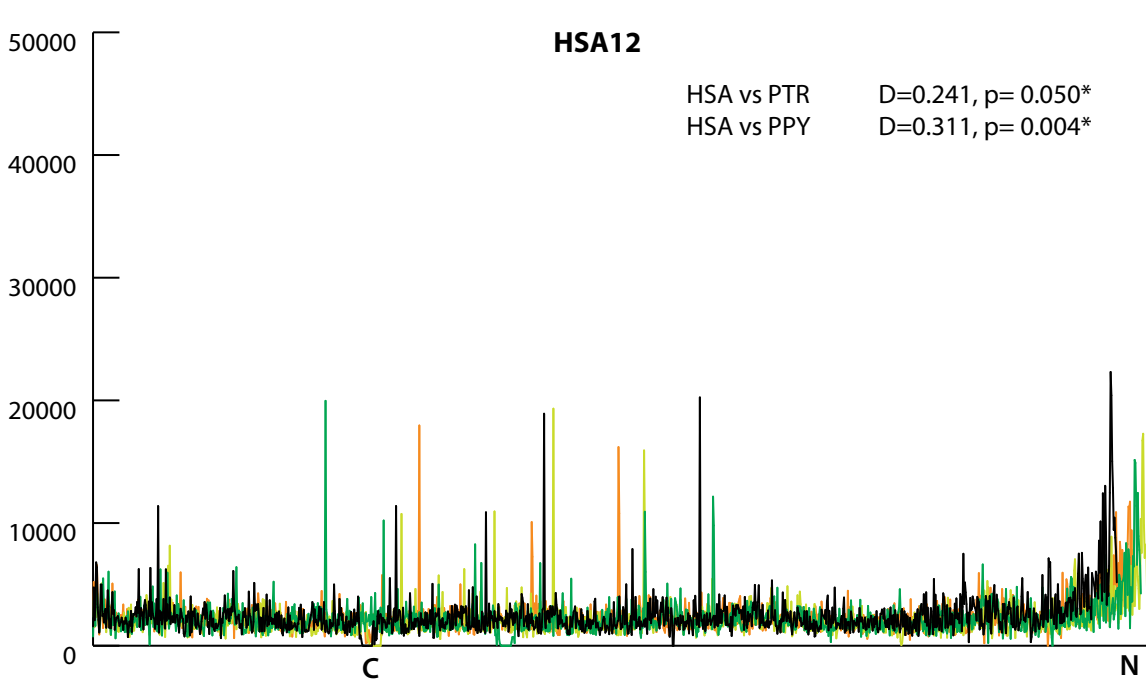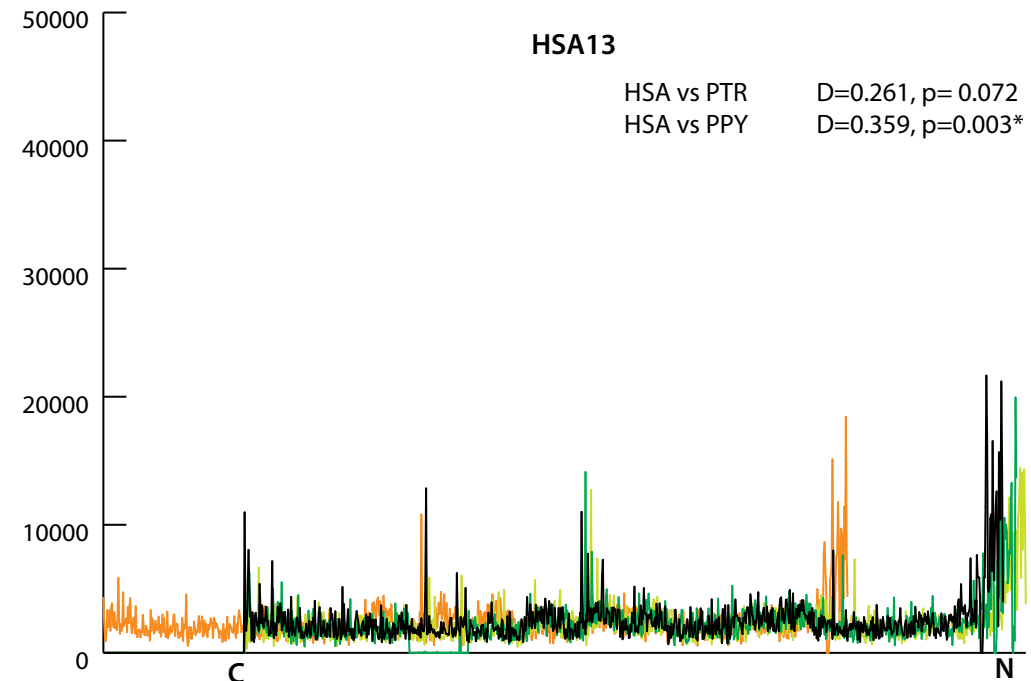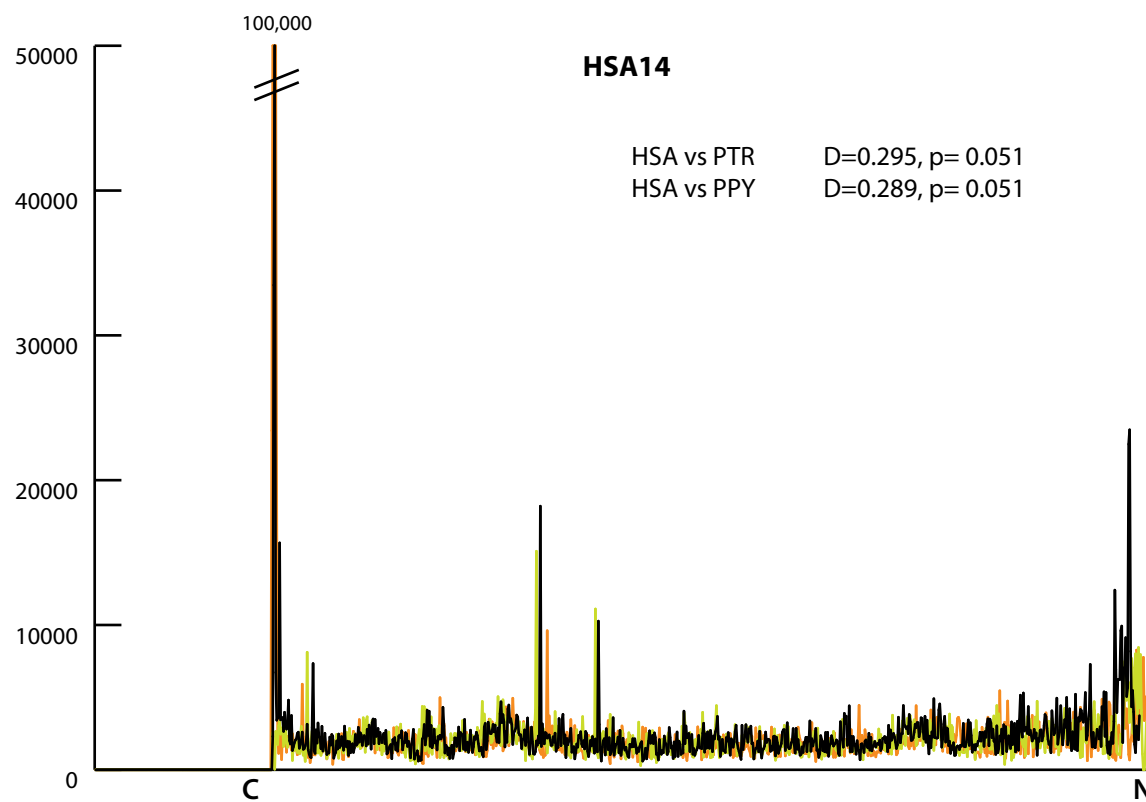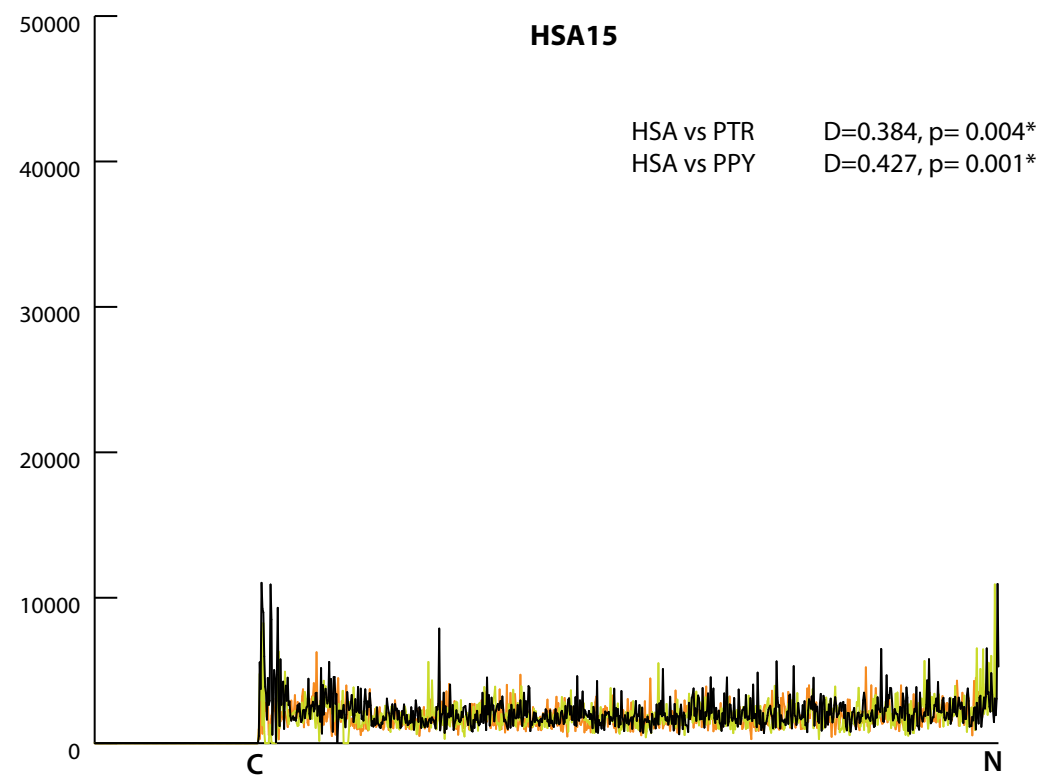

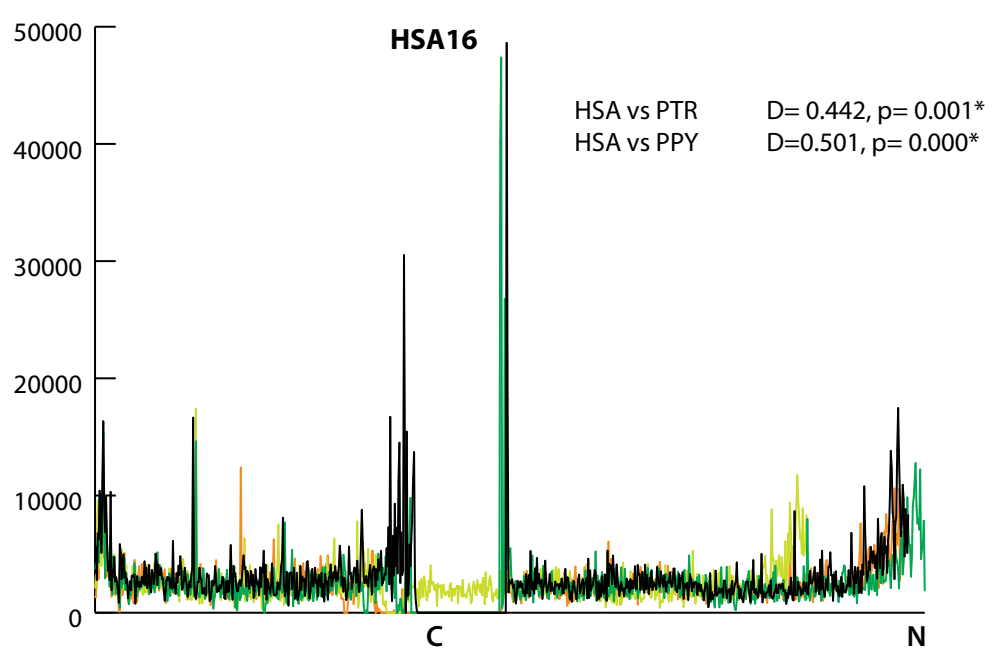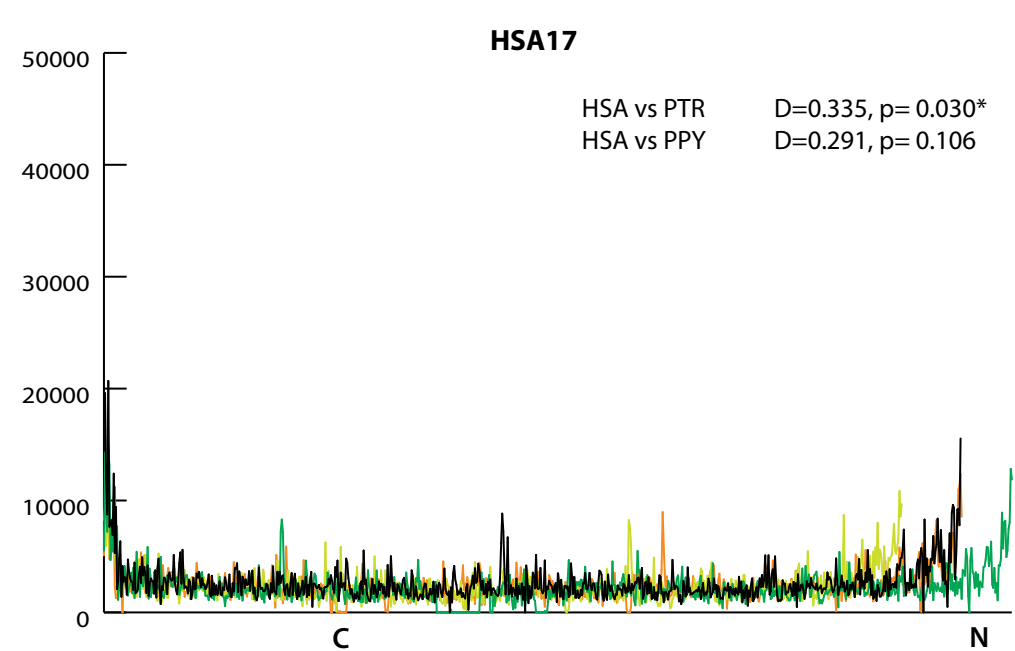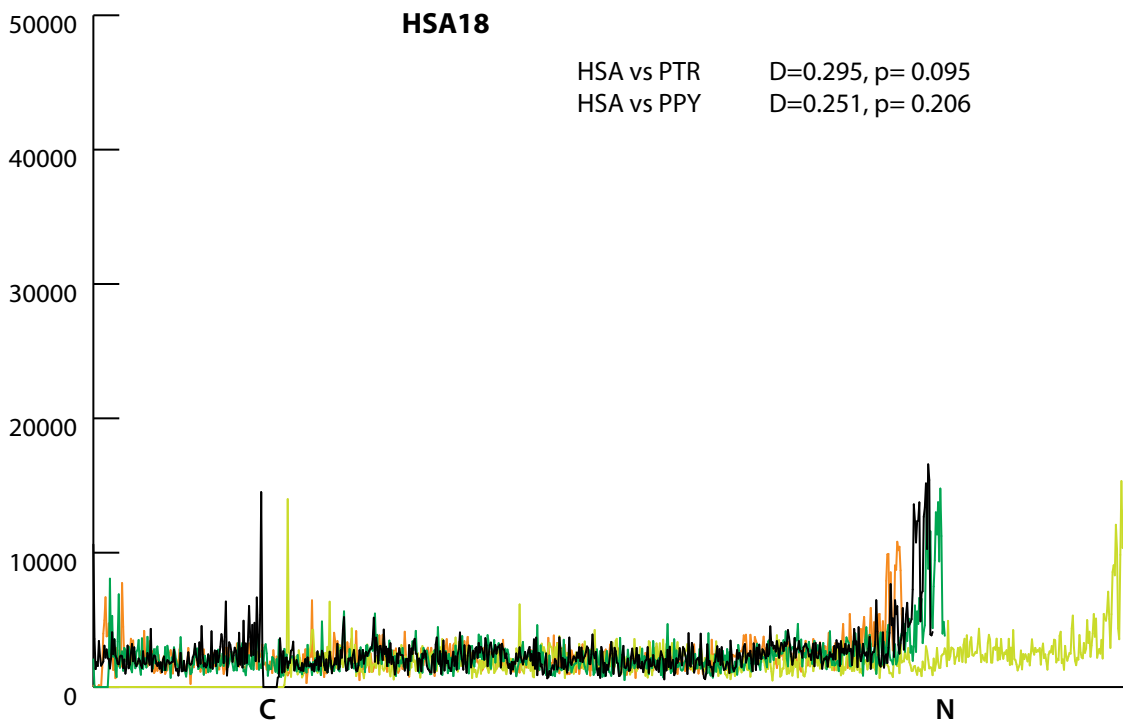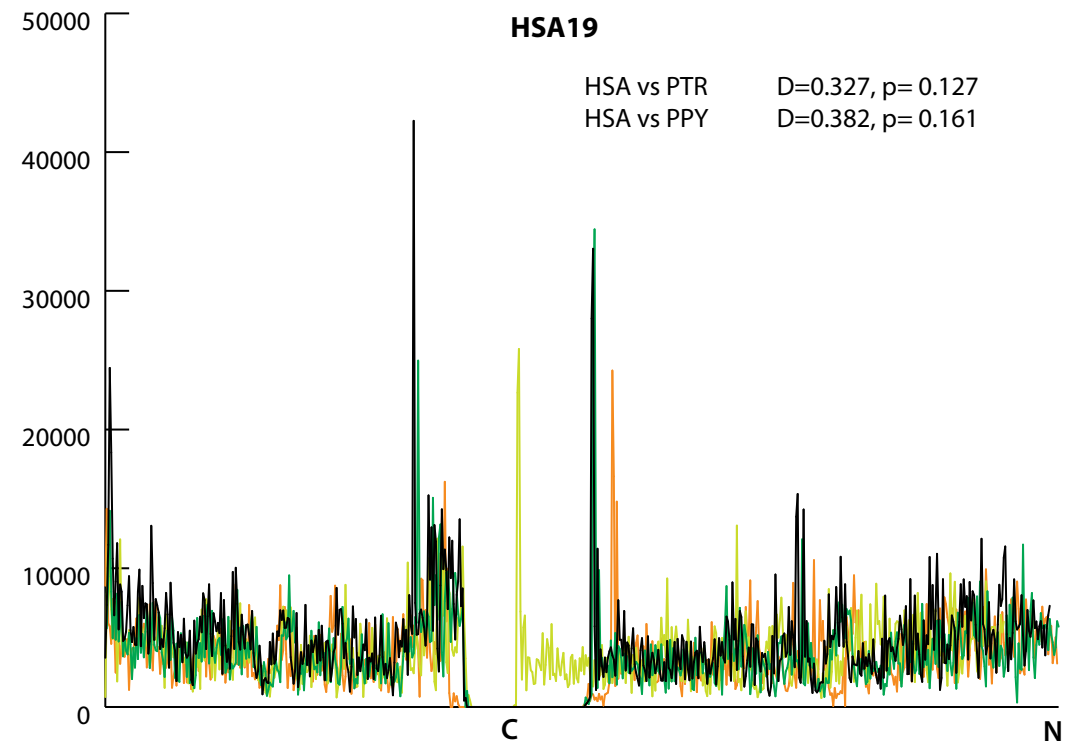

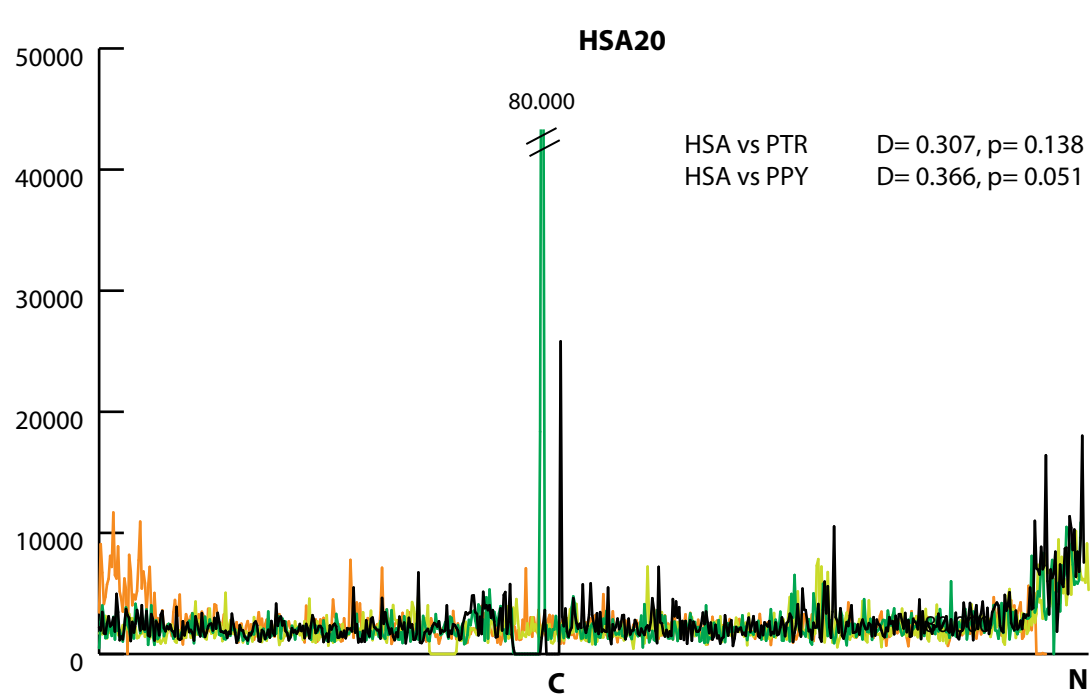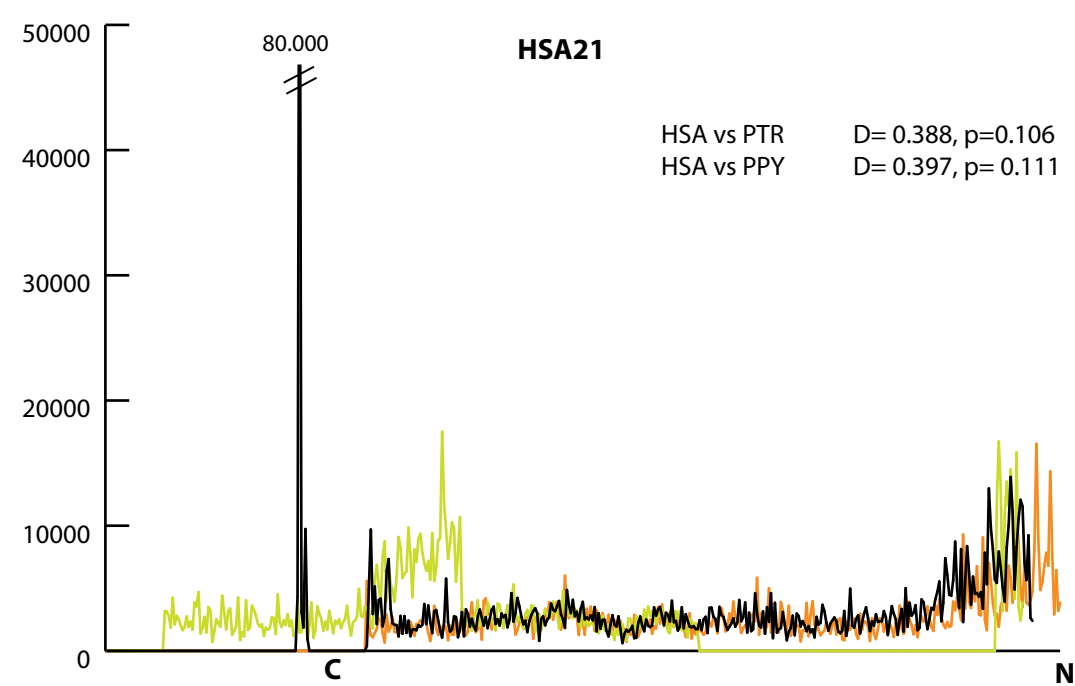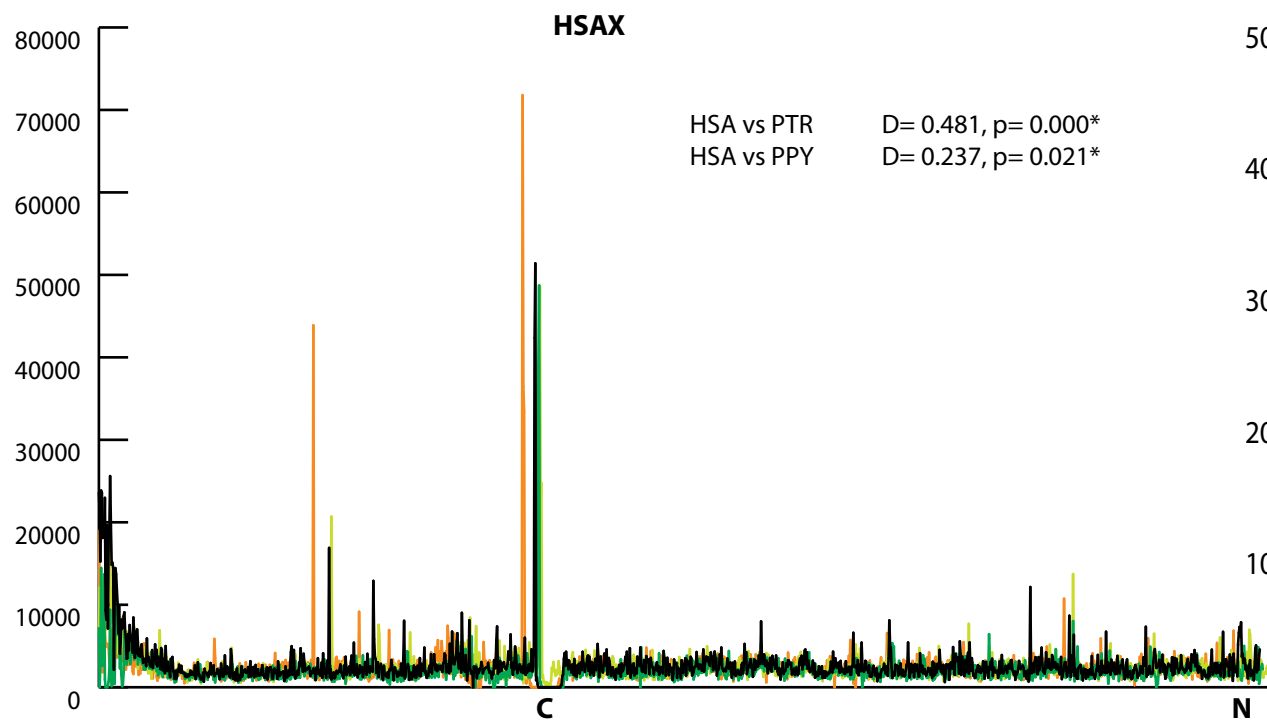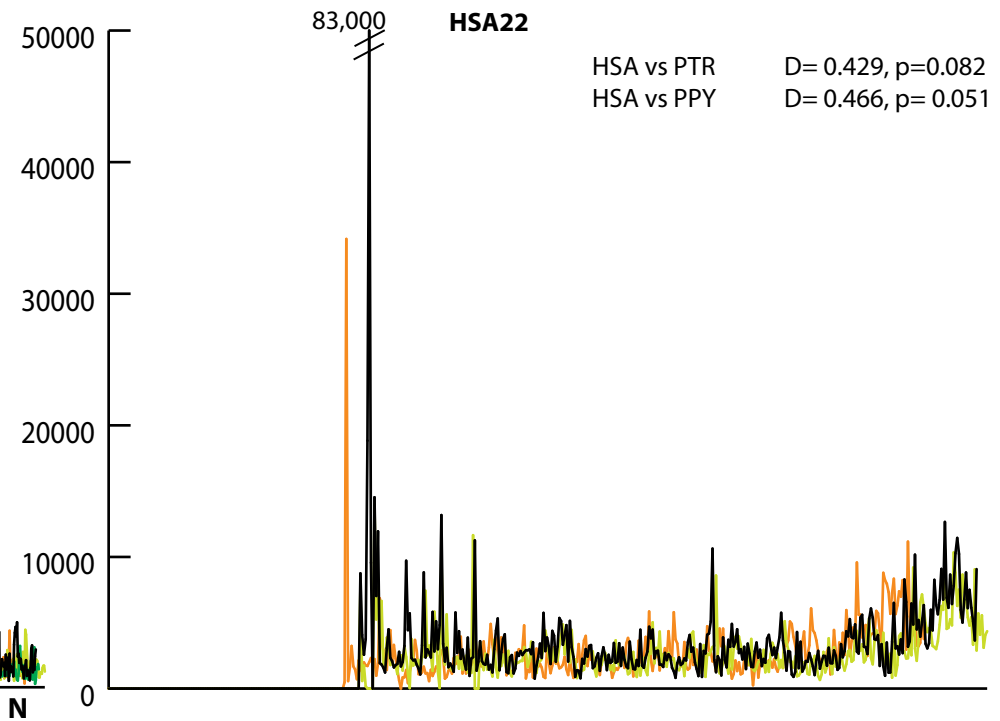

Supplement: File S2 — Tandem repeat content (bp) in non-overlapping 100 kb windows. For each chromosome, the tandem repeat distribution for human (black), chimpanzee (dark green), orangutan (light green) and macaque (orange) is shown. In each case, the Spearman's p test comparing chimpanzee (PTR), orangutan (PPY) and macaque (MMU) with human (HSA) is indicated. C: centromere, N: distal telomere. * Statistically significant p-value <0.0001. (PDF) [file pone.0027239.s002.pdf]
